# Identification of a Bromodomain‐like Region in 15‐Lipoxygenase‐1 Explains Its Nuclear Localization

**DOI:** 10.1002/anie.202106968

**Published:** 2021-08-31

**Authors:** Deng Chen, Zhangping Xiao, Hao Guo, Dea Gogishvili, Rita Setroikromo, Petra E. van der Wouden, Frank J. Dekker

**Affiliations:** ^1^ Department Chemical and Pharmaceutical Biology Groningen Research Institute of Pharmacy (GRIP) University of Groningen Antonius Deusinglaan 1 9713 AV Groningen The Netherlands

**Keywords:** activity-based probes, bromodomains, inhibitors, lipoxygenases, oxidoreductases

## Abstract

Lipoxygenase (LOX) activity provides oxidative lipid metabolites, which are involved in inflammatory disorders and tumorigenesis. Activity‐based probes to detect the activity of LOX enzymes in their cellular context provide opportunities to explore LOX biology and LOX inhibition. Here, we developed Labelox B as a potent covalent LOX inhibitor for one‐step activity‐based labeling of proteins with LOX activity. Labelox B was used to establish an ELISA‐based assay for affinity capture and antibody‐based detection of specific LOX isoenzymes. Moreover, Labelox B enabled efficient activity‐based labeling of endogenous LOXs in living cells. LOX proved to localize in the nucleus, which was rationalized by identification of a functional bromodomain‐like consensus motif in 15‐LOX‐1. This indicates that 15‐LOX‐1 is not only involved in oxidative lipid metabolism, but also in chromatin binding, which suggests a potential role in chromatin modifications.

## Graphical abstract

Activity‐based labeling of lipoxygenase (LOX) activity using the novel one‐step labeling probe **Labelox B** enables detection of isoenzyme inhibitory selectivity in an ELISA‐based assay and visualization of the localization of LOX activity in living cells. The observed nuclear localization was rationalized by identification of a bromodomain‐like region in the 15‐LOX‐1 isoenzyme.

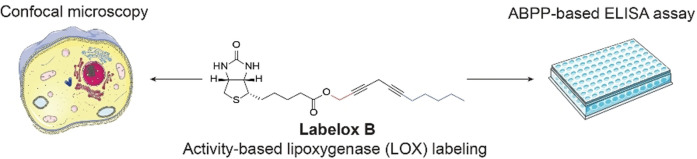

## Introduction

Small molecule modulation of enzyme activity is an important strategy in drug discovery. However, measurement of the activity of specific isoenzymes from the relevant endogenous sources remains challenging. This is particularly true for Lipoxygenases (LOXs), which is a group of enzymes known to be involved in oxidative metabolism of polyunsaturated fatty acids (PUFAs),^[1]^ such as arachidonic acid (AA), and linoleic acid (LA). In humans, LOXs are classified according to the position of O_2_ insertion in AA as 5‐LOX, 12‐LOX or 15‐LOX.^[2]^ LOX activity provides a range of products, such as hydroperoxyl eicosatetraenoic acids (HPETEs), hydroxy eicosatetraenoic acids (HETEs), and leukotrienes (LTs). The activity of LOX enzymes is investigated by changes in the composition of the metabolites resulting from their activity. The LOX lipid metabolites play key roles in various physiological processes involved in inflammatory diseases,^[3]^ cancers and other disease.[^4^, ^5^, ^6^, ^7^] Therefore, small molecule modulation of LOX activity has been explored in drug discovery. However, until now, it remained difficult to investigate the activity and inhibition of specific endogenous LOX isoenzymes, which limits progress in drug discovery.

Currently, there are several methods available to study LOX activity. A common strategy is the identification and quantification of metabolites that originate from LOX activity.[^8^, ^9^] Despite its power, this method does not enable distinction of the activity of LOX enzymes with similar substrate preferences, such as 15‐LOX‐1 and 15‐LOX‐2.^[10]^ Moreover, it does not allow to distinguish the subcellular localization of LOX activity. Nevertheless, the subcellular localization is important for LOX activity, as exemplified by the activity of 5‐LOX. Upon cell stimulation, 5‐LOX traffics and binds to 5‐lipoxygenase‐activating protein (FLAP), which is an integral nuclear envelope protein essential for 5‐LOX mediated leukotriene A4 (LTA4) production.^[11]^ Expounding the relationship between LOXs intracellular localization and activity would promote the understanding of LOX functions. Moreover, the possibility to identify the inhibitory selectivity among endogenous LOX isoenzymes will facilitate our understanding of LOX inhibition and will thus facilitate drug discovery. Therefore, we aim to develop novel tools to investigate the activity of endogenous LOX isoenzymes to advance LOX‐oriented research and drug discovery.

In past decades, activity‐based protein profiling (ABPP) has been employed as a powerful tool^[12]^ facilitating studies on the functions of enzymes in their cellular context^[13]^ and investigations of small molecule inhibitors.^[14]^ Although ABPP of oxidoreductases gained relatively little attention, several ABPs have been developed for this group of enzymes,^[15]^ such as cytochrome P450,^[16]^ monoamine oxidases^[17]^ and flavin monooxygenases.^[18]^ Nevertheless, oxidoreductases remain challenging targets for ABPs. Previously, our group developed the first activity‐based probe (ABP) for enzymes with lipoxygenase activity.^[19]^ The probe **N144** enables two‐step labeling of lipoxygenase activity by the use of a bis‐alkyne as a lipoxygenase reactive functionality, and an alkene functionality as the reactive tag for subsequent biotinylation of alkene labeled proteins. However, the two‐step labeling proved to be inconvenient and not compatible with cellular imaging studies. Here, we developed a probe for one‐step labeling ABP of LOX activity that we employed to investigate isoenzyme selective inhibition by small molecule inhibitors and to visualize the subcellular localization of LOX activity. The observed 15‐LOX‐1 localization in the nucleus enabled identification of a bromodomain‐like region in 15‐LOX‐1 that directly interacts with acetylated histone H3. The presence of an epigenetic reader domain in 15‐LOX‐1 could also play a role in epigenetics and chromatin remodeling.

## Results

**Figure 1:**
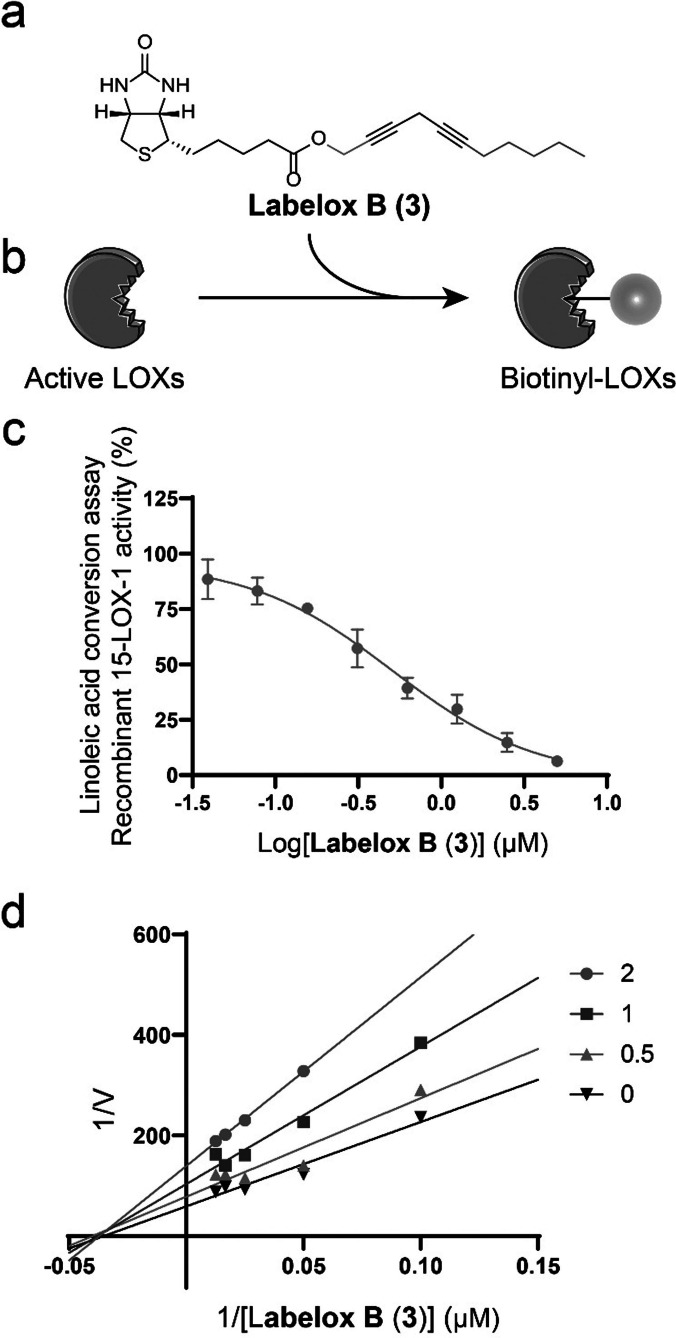
Development and characterization of the activity‐based probe for lipoxygenases. a) Chemical structure of **Labelox B** (**3**). b) Schematic representation of ABPs covalently labeling LOXs. c) IC_50_ curve for **Labelox B** (**3**). Data are mean±SME. *n*≥3 independent experiments. d) Lineweaver–Burk plots for 15‐LOX‐1 inhibition by **Labelox B** (**3**). Data are mean, *n*=2 independent experiments.

**Labelox B (3) is a potent covalent inhibitor for 15‐LOX‐1**. We developed a concise two‐step synthesis route to prepare probes for one‐step activity‐based labeling of LOX, which contains a bis‐alkyne part for LOX labeling and a biotin tag for detection. In brief, an alkyne‐alcohol was linked to propargyl halide (Cl or Br) using a CuI‐catalyzed Sonogashira cross‐coupling reaction followed by biotinylation of the alcohol using EDCI and HOBt as reagents to provide the desired biotinylated bis‐alkyne probes. The biotinylated bis‐alkynes were subjected to 15‐LOX‐1 binding studies using a 15‐LOX‐1 activity assay based on the UV‐absorbance change for linoleic acid conversion (Figure 1 a). The potency was estimated using inhibiting concentration 50 % (IC_50_) values determined after fixed pre‐incubation times. Compound **1** proved to have an intermediate potency with an IC_50_ around 40 μM. The LOX inhibitory potency was lost upon methyl branching of the propargyl methylene linker as found in compound **2**. Also, extending the propargyl methylene linker to two or three carbon atoms, as found in **4** and **5**, provided a loss of LOX inhibitory potency. Extending the methylene linker to 4 carbon atoms, as found in compound **7**, provided a regain in potency. Next, we evaluated the effect of the extension of the terminal ethyl substitution of inhibitor **1**. Extending the terminal ethyl to a pentyl, as found in inhibitor **3** provided a major improvement of the IC_50_ to reach 0.62 μM (Figure 1 c and Table 1). Combination of the terminal pentyl with extensions of the propargyl methyl linker as found in **6** and **8** did not improve the potency. To further assess the mode of binding, we generated Lineweaver–Burk plots for inhibitor **3** (Figure 1 d) as well as inhibitors **1**, **7**, and **8** (Figure S1a), which indicate non‐competitive LOX inhibition in which the *K*_m_ is constant and the V_max_ decreases with the inhibitor concentration. These observations are consistent with our observations for the previously described bis‐alkyne probe **N144**, which also showed non‐competitive inhibition on 15‐LOX‐1.^[19]^

**Table 1:** Structure–activity relationship studies for inhibition of 15‐LOX‐1 activity.

Structure	Probe	R	IC_50_ [μM]			*k*_i_ [min^−1^]	*K*_i_ [μM]
			Value	+ Error	− Error		
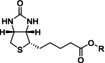	**1**	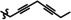	38.2	9.2	7.4	0.0136±0.0061	116.44±19.48
	**2**	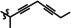	>100	N/A	N/A	N/A	N/A
	**Labelox B** (**3**)	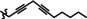	0.6	0.2	0.1	0.0191±0.0038	0.50±0.14
	**4**	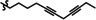	11.1	2.3	1.9	0.0093±0.0031	16.58±3.29
	**5**	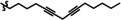	19.8	3.6	3.0	0.0066±0.0047	38.72±6.33
	**6**	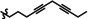	>100	N/A	N/A	N/A	N/A
	**7**	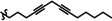	>100	N/A	N/A	N/A	N/A
	**8**	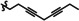	>100	N/A	N/A	N/A	N/A
	**9**	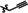	>100	N/A	N/A	N/A	N/A
	**10**	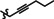	>100	N/A	N/A	N/A	N/A

Subsequently, Kitz‐Wilson analysis was performed to distinguish the equilibrium binding constant (*K*_i_) from the rate of covalent inactivation (*k*_i_). The different bis‐alkynes provided remarkably constant *k*_i_’s that varied by a factor 3 or less, whereas the *K*_i_’s varied by a factor 200 (Table 1). Remarkably, inhibitor **3** provided a *K*_i_ of 0.5 μM, which is a 100‐fold improvement compared to inhibitor **1**. Also comparing the *K*_i_ of **3** to the *K*_i_ of the previously reported probe **N144**, indicates an improvement by one or two orders of magnitude. Improved non‐covalent binding is an important characteristic for activity‐based labeling because it implies improved specific recognition properties to the target of interest. Therefore, inhibitor **3** has been used for further development of a probe for activity‐based labeling of lipoxygenase activity with 15‐LOX‐1 as a reference enzyme. For convenient discussion, we named inhibitor **3**, **“Labelox B**”.

**Figure 2:**
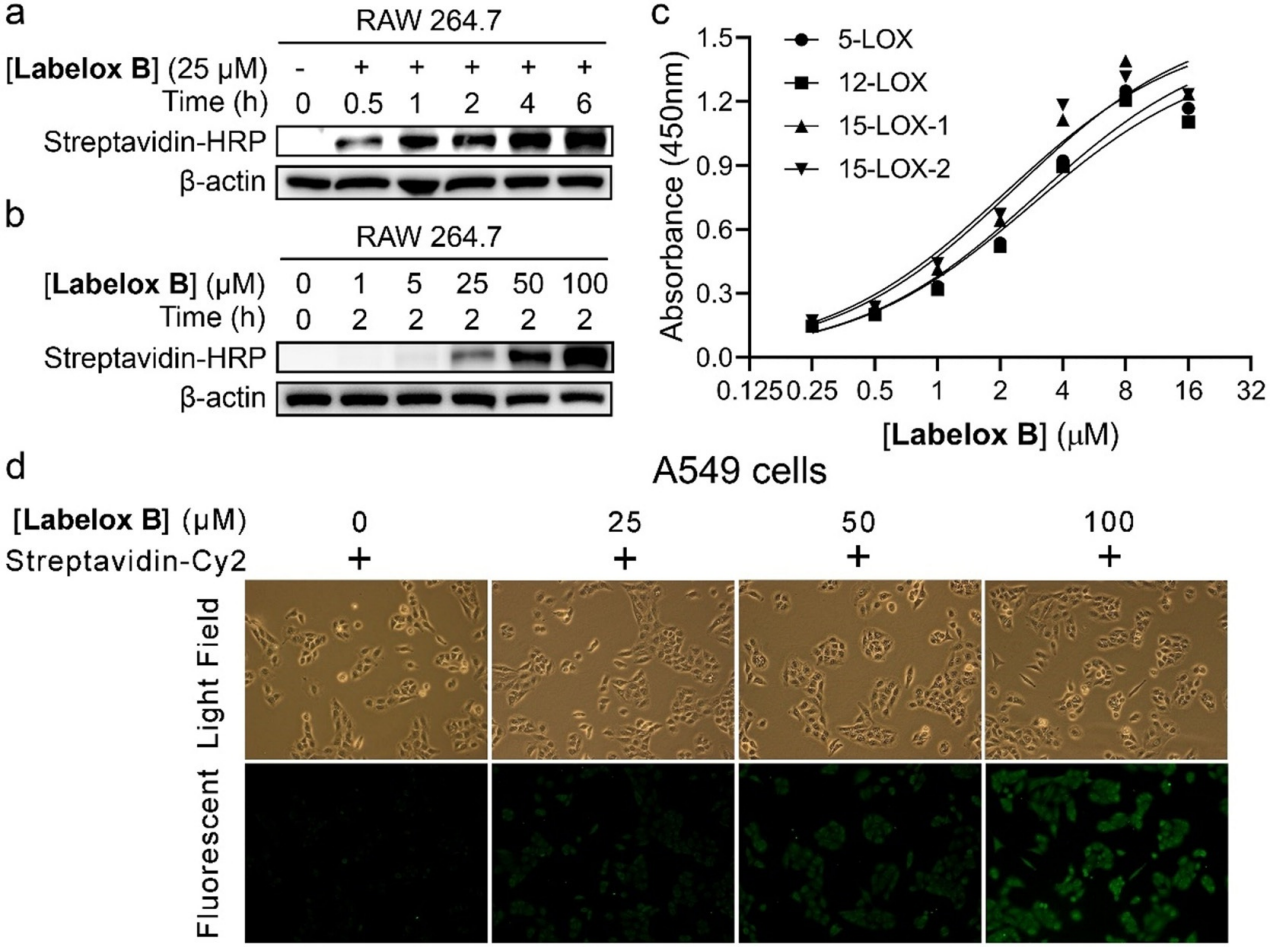
**Labelox B** labels endogenous LOXs in a dose‐ and time‐dependent manner. a, b) Western blot analysis of **Labelox B** labeled LOXs in intact RAW 264.7 for indicated doses and time points. Biotinylation was visualized by western blotting, and β‐actin was employed as a loading control. c) Sandwich ELISA assays to capture lipoxygenase isoenzymes and to monitor biotinylation by **Labelox B**. Samples excluding **Labelox B** were used as negative controls. Data are mean±s.d., *n*≥3 independent experiments. d) Dose‐dependent labeling by **Labelox B** in living cells. A549 cells were treated with **Labelox B** for 1 h at 37 °C. Then, the cells were fixed and permeabilized with methanol. The labeled LOXs were visualized with Cy2‐conjugated streptavidin. The pictures were captured by a Leica DM4000b fluorescence microscope.

**Labelox B efficiently labels endogenous LOXs**. Lipoxygenase labeling by **Labelox B** was investigated using cell‐based assays. As a first step, labeling efficiency was estimated in intact RAW 264.7 macrophage cells that were treated with **Labelox B** in concentrations ranging between 1 μM and 100 μM with incubation times between 0.5 h and 6 h. Western blotting indicated that **Labelox B** labeled proteins with a size corresponding to the mass of endogenous LOXs in a dose and time‐dependent manner (Figure 2 a,b). This indicates that **Labelox B** is sufficiently cell‐permeable to label endogenous LOX in intact cells effectively in half an hour at a concentration of 25 μM. As a next step, a sandwich ELISA assay was established in order to find proof that probe **Labelox B** is indeed labeling proteins with lipoxygenase enzyme activity. Towards this aim, A549 cell lysates were treated with sub‐micromolar to micromolar concentrations of **Labelox B**. Subsequently, the endogenous LOXs were captured on an anti‐LOX antibody precoated 96‐well plate followed by detection of **Labelox B** mediated biotinylation using HRP‐conjugated streptavidin. The ELISA data showed that **Labelox B** efficiently labeled four lipoxygenase isoenzymes, namely 5‐LOX, 12‐LOX, 15‐LOX‐1, and 15‐LOX‐2, with an effective concentration 50 % (EC_50_) around 3.5 μM (Figure 2 c), thus creating an ABP‐based ELISA assay for all LOX isoenzymes. Furthermore, probe **Labelox B** enabled visualization of localized LOX activity in living cells (Figure 2 c). A 549 cells were treated with different doses of **Labelox B**. After fixation and permeabilization, biotin‐tagged LOXs were visualized by Cy2‐conjugated streptavidin. The fluorescence intensity increased with increasing doses of **Labelox B** and labeling was observed throughout the cells in the cytoplasm and the nucleus of A549. Altogether, this indicates that **Labelox B** labels cellular proteins in a time and concentration‐dependent manner, that it labels all human lipoxygenase isoenzymes and that labeling can be visualized cells using fluorescence microscopy.

**Figure 3:**
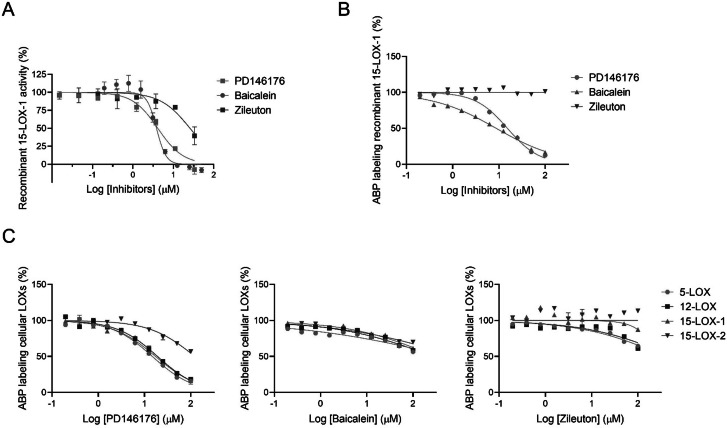
ABP‐based ELISA assay is a fast and convenient method to estimate inhibitory selectivity among LOX isoenzymes. A) Inhibition of recombinant 15‐LOX‐1 catalyzed linoleic acid conversion in a UV absorbance assay. B) Inhibition of recombinant 15‐LOX‐1 ABP‐labeling using the ABP‐based ELISA assay. C) ABP‐based ELISA assay with A549 cell lysate reveals the distinct specificity of LOX inhibitors among LOX isoenzymes. A549 cell lysate was treated with a serial dilution of the respective inhibitor. Subsequently, samples were labeled by treatment with 1 μM of **Labelox B** for 1 min. EDTA then efficiently stopped the reaction (Figure S5). Data are mean±s.d., *n*≥3 independent experiments.

**Assessment of lipoxygenase inhibitor selectivity by the ABP‐based ELISA**. Currently, the analysis of LOX metabolites is a common strategy to determine the selectivity of LOX inhibitors. However, metabolite analysis does not enable the distinction of 15‐LOX‐1 and 15‐LOX‐2 activity. Here, we employed the ABP‐based ELISA assay to study the specificity of LOX inhibitors among LOX isoenzymes. Three well‐known LOX inhibitors were employed to gain insight in the performance of this assay. The first inhibitor is Baicalein, which is a redox inhibitor for LOXs[^20^, ^21^] with an IC_50_ of less than 4 μM in vitro. The second inhibitor is PD146176, which is a 15‐LOX inhibitor^[22]^ with micromolar potency. The third inhibitor is Zileuton,^[23]^ which is a 5‐LOX inhibitor that is currently on market for the maintenance treatment of asthma. The inhibitors were evaluated in the linoleic acid conversion assay using recombinant 15‐LOX‐1 produced in *E.coli* (Figure 3 A). In this assay, PD146176 provided inhibition with IC_50_ around 4 μM and a Hill‐slope of 1, whereas Baicalein provided a similar IC_50_ value with a much higher Hill‐slope. We note that Hill‐slopes deviating from 1 indicate binding behavior deviating from a 1:1 non‐covalent interaction. For Baicalein, the deviating Hill‐slope might be connected to redox inhibition of 15‐LOX‐1 activity. Inhibitors PD147176, Baicalein, and Zileuton were also evaluated in the ABP‐based ELISA assay conducted with recombinant 15‐LOX‐1 (Figure 3 B). We found that PD146176 efficiently attenuated the **Labelox B** mediated LOXs labeling with an IC_50_ around 15 μM and a Hill slope around 1. Baicalein also attenuated 15‐LOX‐1 activity in the ABP‐based ELISA assay with a similar IC_50_ value similar to the linoleic acid conversion assay, however here the Hill‐slope proved to be lower than 1. We anticipate that 15‐LOX‐1 inhibition by redox activity rather than non‐covalent interaction causes a dissimilarity between both assays.

Subsequently, we employed the ABP‐based ELISA assay for cellular lipoxygenase enzymes by the use of isoenzyme selective antibodies to study the inhibitory potency of lipoxygenase inhibitors. The results indicate that PD146176 inhibited 5‐LOX, 12‐LOX, and 15‐LOX‐1 with IC_50_ values of around 17 μM, while 15‐LOX‐2 has an IC_50_ greater than 100 μM (Figure 3 C, left). The Hill‐slope for inhibition of 5‐LOX, 12‐LOX and 15‐LOX‐1 by PD146176 is one (Table S1), which suggests that binding between PD146176 and these isoenzymes is a 1:1 binding event. The IC_50_ and Hill‐slope for PD146176 mediated inhibition of cellular 15‐LOX‐1 activity is similar to that of recombinant 15‐LOX‐1 (IC_50_=15 μM). Furthermore, this analysis indicated that PD146176 inhibited ABP labeling of 5‐LOX, 12‐LOX, and 15‐LOX‐1 with equal potency, whereas the ABP labeling of 15‐LOX‐2 is much less affected. This indicates that PD146176 is selective among 15‐LOX‐1 and 15‐LOX‐2 but not among 5‐LOX, 12‐LOX, and 15‐LOX‐1. In contrast to PD146176, Baicalein has less influence on ABP‐labeling of cellular 15‐LOX‐1 compared to recombinant 15‐LOX‐1 and no selectivity among the cellular LOX enzymes was observed (Figure 3 C, middle). This might be caused by the redox inhibitory mechanism, which might be less effective in a cellular context in comparison to the recombinant enzyme. For the 5‐LOX inhibitor, Zileuton,^[23]^ we found inhibition of 5‐LOX and 12‐LOX at high micromolar concentrations, whereas little or no effect on 15‐LOX‐1 and 15‐LOX‐2 was observed(Figure 3 C, right). Thus the ABP‐based assay enables determination of the potency and selectivity of inhibitors among the endogenous lipoxygenase isoenzymes.

**Lipopolysaccharide stimulates nuclear LOX accumulation**. The ability of probe **Labelox B** to perform activity‐based labeling of endogenous LOX isoenzymes in mammalian cells (Figure 2 c) motivated us to investigate the utility to localize cellular LOX activity using fluorescence microscopy. In this study we note however, that we can not exclude the possibility that **Labelox B** also labels non‐LOX proteins and therefore we refer in this part to putatively active LOX. By comparing immunostaining of LOX isoenzymes with activity‐based labeling of total LOX activity, we aimed to gain more insight in cellular localizations of LOX activity. Previously, it has been shown that LOX isoenzymes are distributed over cytoplasm and nuclei.[^11^, ^24^, ^25^, ^26^] LOX enzyme activity has been described to occur mainly on the nuclear membrane, where arachidonic acid is released to act as a substrate for LOX mediated oxygenation. Here, we investigated how the localization and activation of cellular lipoxygenases changes under the influence of lipopolysaccharide(LPS) as a stimulus in RAW 264.7 macrophages and in A549 cells. We employed confocal microscopy to colocalize active LOXs labeled with **Labelox B** and LOX isoenzymes labeled by immunostaining.

**Figure 4:**
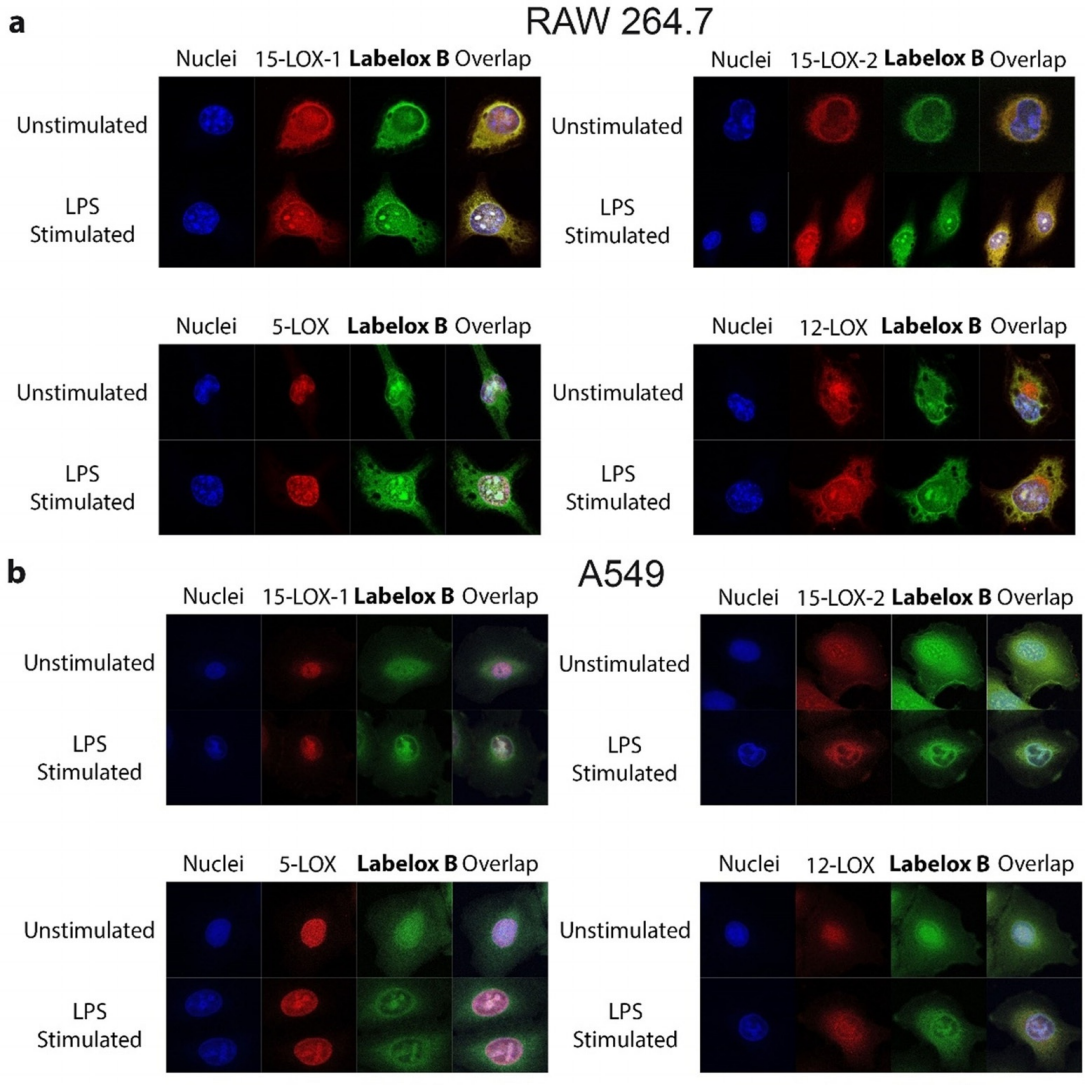
Colocalization analysis of active LOXs and total LOXs in a) RAW264.7 and b) A549. Most of **Labelox B** labeled LOXs are colocalized with the antibody labeled LOXs, indicating that most of the lipoxygenases in cells are catalytically active. However, in RAW264.7 cells, a large number of 12‐LOXs are inactive. LPS stimulation promotes the change of inactive 12‐LOX to a catalytically active state. Pictures are representative of three independent experiments.

The confocal pictures demonstrated that LOX antibody‐ and **Labelox B** labeled LOXs were mainly colocalized, suggesting that **Labelox B** was sufficient to visualize active LOXs in living cells. Under non‐stimulated conditions, the isoenzymes 12‐LOX, 15‐LOX‐1, and 15‐LOX‐2 were localized in both cytoplasm and nuclei of RAW264.7 and A549 cells, whereas the 5‐LOX localization seemed to be restricted to the nucleus. Some interesting observations were made. First, in RAW264.7 cells, a relatively large amount of 12‐LOX was observed that could not be colocalized with **Labelox B** labeling, whereas other LOX isoenzymes do also not seem to accumulate in the cytoplasm (Figure 4 a). This could indicate a pool of inactive 12‐LOX in the cytoplasm that disappears upon LPS stimulation. Next, we observed a change in localization of the LOX isoenzymes that was the clearest in RAW264.7 macrophages. Upon stimulation, the localization becomes more pronounced in the nuclear membrane and remarkably also on distinct locations in the nucleus. This staining pattern for LOX localization has been observed before for 5‐LOX in macrophages.^[11]^ Importantly, the change in localization of all LOX isoenzymes colocalized with a change in activity‐based LOX labeling with probe **Labelox B** (Figure 4). This indicates that the LOX isoenzymes are activated at the nuclear membranes as described in literature before.[^11^, ^24^] However, LOX enzyme activity at distinct nuclear localizations have not been described before. We were intrigued by this finding and set out to rationalize the LOX localization in the nucleus.

**Figure 5:**
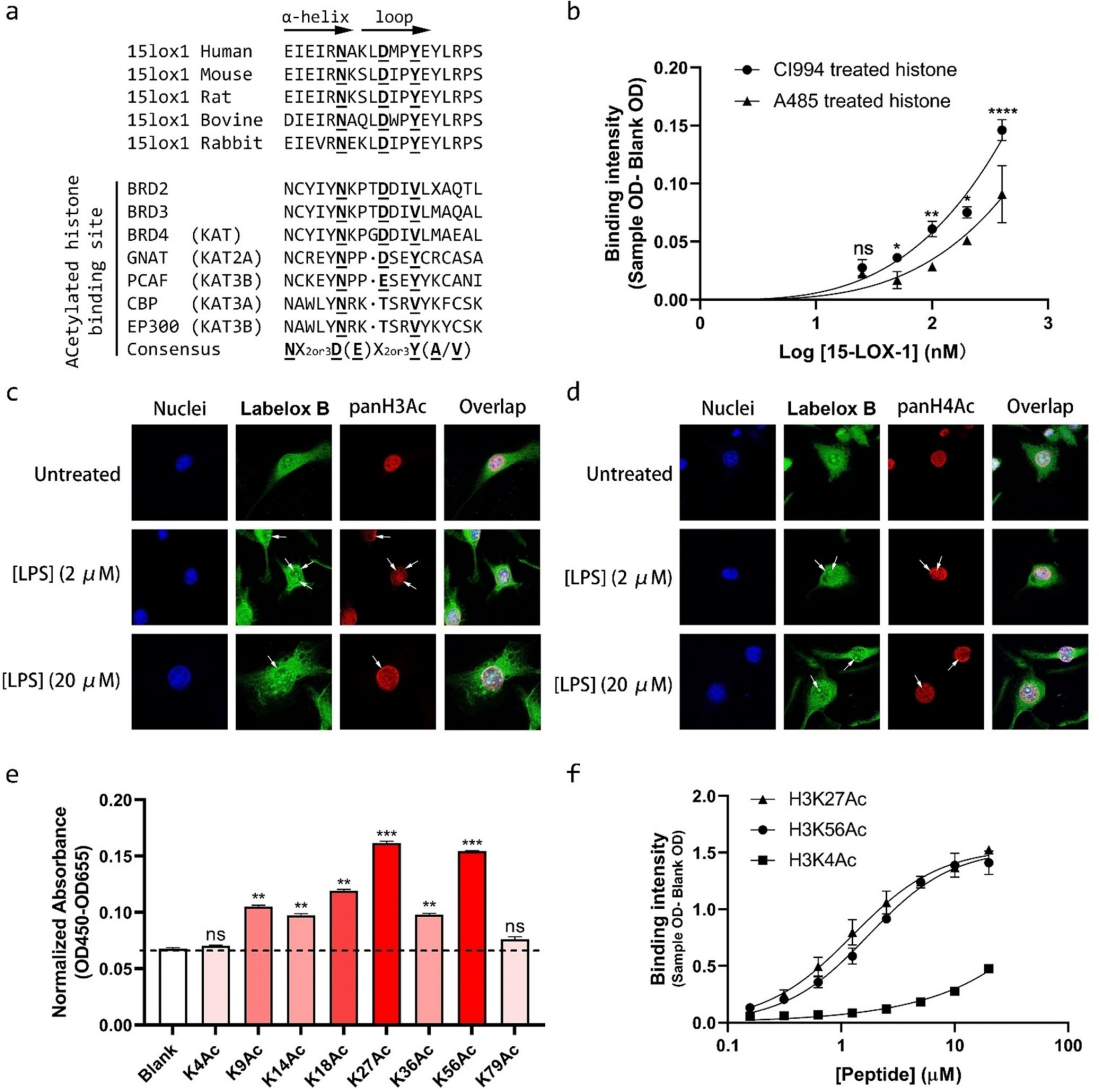
15‐LOX‐1 contains a bromodomain‐like region that specifically interacts with histone H3K27Ac and H3K56Ac, but not H3K4Ac or H3K79Ac. a) The consensus motifs of a bromodomain, Nx2or3D(E)x2or3Y(A/V), are conserved in many other bromodomains containing proteins as well as in 15‐LOX‐1 across mammalian species. b) ELISA assay for binding of 15‐LOX‐1 to histone coated plates with various levels of acetylation. Data are mean±s.d., *P<0.03, **P<0.003, ****P<0.0001, *n*≥3 independent experiments, Two‐way ANOVA. c, d) Confocal fluorescence microscopy analysis showed that **Labelox B**‐labeled LOXs colocalized with acetylated histone H3 and H4. Pictures are representative of three independent experiments. e) Acetylated histone H3 peptide array identified specific residues interacting with 15‐LOX‐1. Data are mean±s.d., **P<0.003, ***P<0.0003, *n*=3 readouts, One‐way ANOVA. f) ELISA showed distinct binding affinity between 15‐LOX‐1 and diverse H3Ac peptides. Data are mean±SME, *n*≥3 independent experiments.

**15‐LOX‐1 has a bromodomain‐like region that binds to acetylated histones**. In our search for a physiological explanation for the distinct nuclear localization of LOX isoenzymes, we focused on 15‐LOX‐1. The primary sequence of 15‐LOX‐1 was analyzed for epigenetic reader domains based on the presumption that chromatin binding could explain the distinct nuclear localization of 15‐LOX‐1. We identified the consensus motifs for a bromodomain, Nx2or3D(E)x2or3Y(A/V),[^27^, ^28^, ^29^] in the primary structure of 15‐LOX‐1. This bromodomain consensus sequence is also found in other bromodomain containing proteins, such as bromodomain and extra terminal domain (BET) and histone acetyltransferase (HAT) family members (Figure 5 a, lower part). Such consensus residues are also conserved in 15‐LOX‐1 across mammalian species (Figure 5 a, upper part). Among these residues, asparagine is the most essential as it directly interacts with acetylated lysine residues on histones.[^27^, ^28^, ^29^] Based on this sequence analysis, we hypothesized that 15‐LOX‐1 binds to histones depending on their acetylation level. Therefore, we prepared two histone extractions with different levels of total acetylation. Highly acetylated histones were extracted from A549 cells treated with inhibitor CI994, a histone deacetylase inhibitor, whereas lowly acetylated histones were prepared from A549 cells treated with A485, a histone acetyltransferase inhibitor. An ELISA‐binding assay demonstrated that histones with higher acetylation levels had increased 15‐LOX‐1 binding compared to histones with lower acetylation levels, thus supporting the hypothesis that recombinant 15‐LOX‐1 binds acetylated histones (Figure 5 b). Moreover, confocal microscope pictures showed that LOX labeling with probe **Labelox B** colocalized with both acetylated H3 and H4 (Figure 5 c,d). Upon LPS stimulation, more active LOXs were recruited to acetylated H3 and H4, especially at the inner nuclear envelope area (Figure 5 c,d, white arrows). Furthermore, a microarray with acetylated histone H3 peptides was performed to further characterize the binding of 15‐LOX‐1 to acetylated histones. This microarray indicated that H3K27Ac and H3K56Ac are binding to 15‐LOX‐1, whereas H3K4Ac does not bind to 15‐LOX‐1 (Figure 5 e). To confirm the microarray data, a binding study between the respective lysine‐acetylated histone peptides and 15‐LOX‐1 was performed using an ELISA formatted assay. A clear binding curve was observed for binding of the peptides H3K27Ac and H3K56Ac with 15‐LOX‐1 to provide a *K*_d_ of 1.13 and 1.65 μM, respectively. The Hill‐slopes of these binding curves equal 1 (Figure 5 f). For comparison, a binding study to the corresponding non‐acetylated histone H3 peptide showed a lower binding affinity to 15‐LOX‐1 with a *K*_d_ of 22 μM (Figure S6). Another comparison was made by determining the binding affinity of a H3K4Ac peptide towards 15‐LOX‐1 (Figure 5 f). The *K*_d_ for binding of H3K4Ac to 15‐LOX‐1 exceeded the concentration range used in this assay because no saturation of binding was observed at concentration up to 20 μM. Taken together, this indicates that 15‐LOX‐1 has a functional bromodomain‐like sequence that provides binding to H3K27Ac and H3K56Ac acetylated peptides with affinities in the low micromolar range.

## Discussion

Lipoxygenases have classically been recognized as a group of enzymes involved in lipid signaling in inflammatory diseases and oncology.[^3^, ^30^, ^31^] More recently, a connection with ferroptosis has been described.[^32^, ^33^, ^34^, ^35^] Despite their importance in physiology, it remained difficult to study the activity of lipoxygenase isoenzymes in their cellular context.

Here, we described a versatile probe, **Labelox B**, for one‐step ABP‐labeling of LOX isoenzymes. **Labelox B** is a cell membrane permeable ABP‐probe (Figure 3 a, b, and d) that can be employed to investigate intracellular LOX activity and LOX activity in in cell lysates (Figure 3 c). Using this property, an ABP‐based ELISA was established to determine **Labelox B** ABP‐labeling per LOX isoenzyme and the inhibitory selectivity of LOX inhibitor among these LOX isoenzymes. Interestingly, the 12/15‐LOX selective inhibitor PD146176 provided only selectivity among 15‐LOX‐1 and 15‐LOX‐2 but not for 5‐LOX and 12‐LOX. The inhibition of 5‐LOX and 12‐LOX has not been reported before,[^22^, ^26^] whereas the lack of 15‐LOX‐2 inhibition is in line with the literature. The group of Anne et al. reported that PD146176 did not inhibit the formation of 15‐LOXs product formation in neutrophils which express only 15‐LOX‐2.^[10]^ This indicates that PD146176 is selective among 15‐LOX‐1 and 15‐LOX‐2. Also, the lack of inhibitory selectivity of Baicalein among LOX isoenzymes is in line with the literature. Previous enzymatic assays confirmed that Baicalein has potent inhibitory effects on both 5‐, 12‐, and 15‐LOX.[^21^, ^36^] Additionally, the antioxidative activity of Baicalein was confirmed to contribute to the inhibition of other oxidases, such as xanthine oxidase (XO).^[37]^ Besides, Baicalein has potent free radical scavenging effects, especially on superoxide radicals (^.^O_2_^−^).^[37]^ Such effects attenuate oxidative stress and protect cells from reactive oxygen species (ROS).^[38]^ Furthermore, we found that Zileuton inhibits both 5‐LOX and 12‐LOX, but not 15‐LOXs. This also coincides with the previous findings. Zileuton attenuated the production of 5‐HETE, 5‐HEPE, 12‐HETE, but not 15‐HETE.^[39]^ Although we were not able to find studies that directly determined the binding of Zileuton to 5‐LOX, cell‐based assays indicate that Zileuton potently inhibits the biosynthesis of 5‐LOX metabolites, like LTBs and cysteinyl leukotrienes.^[40]^ However, this effect might be attributed to inhibition of arachidonic acid release in addition to direct effects on 5‐LOX.^[40]^ Thus the 5‐LOX inhibition observed in our assay is in line with previous reports on the 5‐LOX inhibitory potency. Taking this together, we conclude that the ABP‐based ELISA assay provides a fast and convenient complementary method to assess the selectivity of LOX inhibitors.

The ABP‐based ELISA assay may provide a better prediction of in vivo potency of LOX inhibitors compared to activity assay on purified recombinant enzymes. This idea is supported by observations made for the LOX inhibitor Baicalein that proved to be much less potent in the ABP‐based ELISA on endogenous lipoxygenase activity (Figure 3 b,c). A similar observation was reported by Lukas et al., who observed that Baicalein less potently rescued cells from ferroptotic cell death compared to PD146176,^[32]^ whereas Baicalein and PD146176 have a comparable IC_50_ for LOX inhibition. Thirdly, the ABP‐based ELISA does not depend on expression and purification of different LOXs isoforms. This, together, with the straightforward two‐step synthetic procedure to synthesize probe **Labelox B** and the one‐step labeling procedure for LOX activity provides a convenient method that can be used complementary to already existing methods.

A limited number of studies discuss the connection between the localization and activity of endogenous LOXs. Christmas et al. reported that only 5‐LOX and not 15‐LOX translocated to the nuclear envelope upon stimulation.^[11]^ Later, Jones et al. identified three nuclear‐localization‐sequence (NLS) in 5‐LOX.[^41^, ^42^] Mutation of each NLS impaired nuclear import of 5‐LOX. Further, Luo et al. observed that the disruption of all three NLS in 5‐LOX eliminated the production of leukotriene B4 (LTB4) in cells, while the arachidonic acid conversion activity of such mutants largely remained as observed in a cell‐free enzymatic assay.^[25]^ The authors argue that this isoenzyme specific translocation plays a role in the production of specific oxygenation products with specific functions.[^11^, ^25^] Our observations are complementary to previous observations. We confirm that cellular stimulation, by LPS, induced accumulation of LOX isoenzymes on the nuclear envelope. This is particularly pronounced for 5‐LOX (Figure 4 a,b). The change in localizations for all LOX isoenzymes overlapped with a change in localization of ABP probe **Labelox B**, which indicates that a change in localization is accompanied by a change in the localization of LOX activity (Figure 4 a).

Application of probe **Labelox B** indicated that LPS stimulation triggered LOX accumulation at the nuclear envelope as well as at distinct locations in the nucleus. This nuclear accumulation of LOX isoenzymes has been observed before. However, it was expected that LOX was not active in the nucleus^[25]^ Remarkably, we observed clear colocalization of probe **Labelox B** labeling with nuclear localization of LOX ABP labeling, thus suggesting that LOX is active in the nucleus. The connection between LOX localization and LOX ABP labeling at distinct localization in the nucleus could indicate a functional role for the LOX isoenzymes in the nucleus. Using sequence comparison and biochemical tools we were able to identify a bromodomain‐like region in 15‐LOX‐1 with low micromolar affinity for specific lysine acetylated histone peptides. These observations are in line with previous observations for bromodomain‐containing proteins that also provide particular epitope preferences and binding constants in the low micromolar range.[^27^, ^43^, ^44^] The *K*_d_ of H4K4Ac against the firstly identified bromodomains of BRD2 and BRD4 are 4.3 and 3.1 μM, respectively.^[45]^ The *K*_d_ of BAZ2B bromodomain‐H3K14Ac pair is 6.3 μM.^[45]^ Even bromodomain‐acetylated peptide interactions with lower affinity values were reported, such as a H3K36Ac peptide interaction with the CREBBP bromodomain, which provides a *K*_d_ of merely 122 μM,^[45]^ whereas the crystal structure^[43]^ clearly demonstrates a specific interaction.

The classical bromodomain contains a continuous four helices (αZ, αA, αB, αC) with two inter‐helical loops (ZA and BC) of variable length and sequence, in which, the ZA loop is likely to determine the acetyl‐lysine binding.^[46]^ The essential consensus motifs, Nx2or3D(E)x2or3Y(A/V), are located in this loop. Crystal structure shows that the acetyl group in a lysine residue forms a conventional hydrogen bond with an asparagine residue located in the ZA loop.[^27^, ^28^, ^29^] Binding of histone peptides to bromodomains is attenuated by the lack of acetylated lysine residues.^[47]^ The observations that lysine acetylated histone peptides have a higher affinity for the putative bromodomain‐like region in 15‐LOX‐1 are in line with these prior observations. Also, identification of the essential loop with the Nx2or3D(E)x2or3Y(A/V) motif in 15‐LOX‐1 (Figure S7) supports the idea that 15‐LOX‐1 contains a functional bromodomain‐like region.

Taken together, we generated probe **Labelox B** for convenient one‐step activity‐based labeling of lipoxygenase enzyme activity. This probe enabled distinction and quantification of ABP‐labeling of LOX isoenzymes in an ELISA assay, which was utilized to determine inhibitory selectivity among LOX isoenzymes. Moreover, **Labelox B** enabled visualization of putatively active endogenous LOXs in cells, which, for the first time, offers a possibility to connect the activity of LOXs to their different intracellular localizations upon treatment with stimuli. The potential of this approach is demonstrated by the remarkable finding that LOX isoenzymes and ABP‐labeling co‐localized on distinct locations in the nucleus. This observation led to the identification of a bromodomain‐like region in 15‐LOX‐1, which rationalizes the observed nuclear localization 15‐LOX‐1. Thus, this study on the one hand provided a novel chemical tool for ABP‐labeling of LOX isoenzymes and on the other hand indicated that 15‐LOX‐1 has a bromodomain‐like region, which suggests a role in chromatin biology.

## Conflict of interest

The authors declare no conflict of interest.

## Supporting information

As a service to our authors and readers, this journal provides supporting information supplied by the authors. Such materials are peer reviewed and may be re‐organized for online delivery, but are not copy‐edited or typeset. Technical support issues arising from supporting information (other than missing files) should be addressed to the authors.

Supporting Information

## References

[1] A. Liavonchanka , I. Feussner , J. Plant Physiol. 2006, 163, 348–357. 16386332 10.1016/j.jplph.2005.11.006

[2] H. Kuhn , Prostaglandins Other Lipid Mediators 2000, 62, 255–270. 10963793 10.1016/s0090-6980(00)00084-8

[3] H. Kühn , V. B. O'Donnell , Prog. Lipid Res. 2006, 45, 334–356. 16678271 10.1016/j.plipres.2006.02.003

[4] Z. Zheng , Y. Li , G. Jin , T. Huang , M. Zou , S. Duan , Biomed. Pharmacother. 2020, 129, 110354. 32540644 10.1016/j.biopha.2020.110354

[5] H. Costa , J. Touma , B. Davoudi , M. Benard , T. Sauer , J. Geisler , K. Vetvik , A. Rahbar , C. Söderberg-Naucler , J. Cancer Res. Clin. Oncol. 2019, 145, 2083–2095. 31203442 10.1007/s00432-019-02946-8 PMC6658585

[6] J. Ghosh , C. E. Myers , Proc. Natl. Acad. Sci. USA 1998, 95, 13182–13187. 9789062 10.1073/pnas.95.22.13182 PMC23752

[7] H. Kuhn , S. Banthiya , K. Van Leyen , Biochim. Biophys. Acta Mol. Cell Biol. Lipids 2015, 1851, 308–330. 10.1016/j.bbalip.2014.10.002 PMC4370320 25316652

[8] K. M. Gauthier , W. B. Campbell , A. J. McNeish , PeerJ 2014, 2, e414. 24949235 10.7717/peerj.414 PMC4060036

[9] N. Wang , X. Zhao , J. Huai , Y. Li , C. Cheng , K. Bi , R. Dai , J. Ethnopharmacol. 2018, 217, 205–211. 29474901 10.1016/j.jep.2018.02.027

[10] A.-S. Archambault , C. Turcotte , C. Martin , V. Provost , M.-C. Larose , C. Laprise , J. Chakir , É. Bissonnette , M. Laviolette , Y. Bossé , N. Flamand , PLoS One 2018, 13, e0202424. 30118527 10.1371/journal.pone.0202424 PMC6097673

[11] P. Christmas , J. W. Fox , S. R. Ursino , R. J. Soberman , J. Biol. Chem. 1999, 274, 25594–25598. 10464294 10.1074/jbc.274.36.25594

[12] N. Jessani , B. F. Cravatt , Curr. Opin. Chem. Biol. 2004, 8, 54–59. 15036157 10.1016/j.cbpa.2003.11.004

[13] K. T. Barglow , B. F. Cravatt , Nat. Methods 2007, 4, 822–827. 17901872 10.1038/nmeth1092

[14] D. A. Bachovchin , S. J. Brown , H. Rosen , B. F. Cravatt , Nat. Biotechnol. 2009, 27, 387–394. 19329999 10.1038/nbt.1531 PMC2709489

[15] R. Fuerst , R. Breinbauer , ChemBioChem 2021, 22, 630–638. 32881211 10.1002/cbic.202000542 PMC7894341

[16] T. T. Talele , J. Med. Chem. 2020, 63, 5625–5663. 32031378 10.1021/acs.jmedchem.9b01617

[17] J. M. Krysiak , J. Kreuzer , P. MacHeroux , A. Hermetter , S. A. Sieber , R. Breinbauer , Angew. Chem. Int. Ed. 2012, 51, 7035–7040; 10.1002/anie.201201955 PMC3470703 22689512

[18] I. P. McCulloch , J. J. La Clair , M. J. Jaremko , M. D. Burkart , ChemBioChem 2016, 17, 1598–1601. 27271974 10.1002/cbic.201600275 PMC5656434

[19] N. Eleftheriadis , S. A. Thee , M. R. H. Zwinderman , N. G. J. Leus , F. J. Dekker , Angew. Chem. Int. Ed. 2016, 55, 12300–12305; 10.1002/anie.201606876 PMC5218545 27612308

[20] P. A. Lapchak , P. Maher , D. Schubert , J. A. Zivin , Neuroscience 2007, 150, 585–591. 17942241 10.1016/j.neuroscience.2007.09.033

[21] J. D. Deschamps , V. A. Kenyon , T. R. Holman , Bioorg. Med. Chem. 2006, 14, 4295–4301. 16500106 10.1016/j.bmc.2006.01.057

[22] T. M. A. Bocan , W. S. Rosebury , S. B. Mueller , S. Kuchera , K. Welch , A. Daugherty , J. A. Cornicelli , Atherosclerosis 1998, 136, 203–216. 9543090 10.1016/s0021-9150(97)00204-9

[23] S. Sinha , M. Doble , S. L. Manju , Bioorg. Med. Chem. 2019, 27, 3745–3759. 31331653 10.1016/j.bmc.2019.06.040

[24] J. W. Woods , J. F. Evans , D. Ethier , S. Scott , P. J. Vickers , L. Hearn , J. A. Heibein , S. Charleson , I. I. Singer , J. Exp. Med. 1993, 178, 1935–1946. 8245774 10.1084/jem.178.6.1935 PMC2191287

[25] M. Luo , S. M. Jones , M. Peters-Golden , T. G. Brock , Proc. Natl. Acad. Sci. USA 2003, 100, 12165–12170. 14530386 10.1073/pnas.2133253100 PMC218730

[26] J. Timár , E. Rásó , B. Döme , L. Li , D. Grignon , D. Nie , K. V. Honn , W. Hagmann , Int. J. Cancer 2000, 87, 37–43. 10861450 10.1002/1097-0215(20000701)87:1<37::aid-ijc6>3.0.co;2-l

[27] T. Umehara , Y. Nakamura , M. K. Jang , K. Nakano , A. Tanaka , K. Ozato , B. Padmanabhan , S. Yokoyama , J. Biol. Chem. 2010, 285, 7610–7618. 20048151 10.1074/jbc.M109.062422 PMC2844208

[28] C. Tallant , E. Valentini , O. Fedorov , L. Overvoorde , F. M. Ferguson , P. Filippakopoulos , D. I. Svergun , S. Knapp , A. Ciulli , Structure 2015, 23, 80–92. 25533489 10.1016/j.str.2014.10.017 PMC4291147

[29] J. Morinière , S. Rousseaux , U. Steuerwald , M. Soler-López , S. Curtet , A. L. Vitte , J. Govin , J. Gaucher , K. Sadoul , D. J. Hart , J. Krijgsveld , S. Khochbin , C. W. Müller , C. Petosa , Nature 2009, 461, 664–668. 19794495 10.1038/nature08397

[30] J. Z. Haeggström , C. D. Funk , Chem. Rev. 2011, 111, 5866–5896. 21936577 10.1021/cr200246d

[31] R. Mashima , T. Okuyama , Redox Biol. 2015, 6, 297–310. 26298204 10.1016/j.redox.2015.08.006 PMC4556770

[32] L. Probst , J. Dächert , B. Schenk , S. Fulda , Biochem. Pharmacol. 2017, 140, 41–52. 28595877 10.1016/j.bcp.2017.06.112

[33] M. Conrad , D. A. Pratt , Nat. Chem. Biol. 2019, 15, 1137–1147. 31740834 10.1038/s41589-019-0408-1

[34] L. Shen , D. Lin , X. Li , H. Wu , C. Lenahan , Y. Pan , W. Xu , Y. Chen , A. Shao , J. Zhang , Front. Cell Dev. Biol. 2020, 8, 1–16. 32760721 10.3389/fcell.2020.00594 PMC7373735

[35] W. S. Yang , K. J. Kim , M. M. Gaschler , M. Patel , M. S. Shchepinov , B. R. Stockwell , Proc. Natl. Acad. Sci. USA 2016, 113, E4966–E4975. 27506793 10.1073/pnas.1603244113 PMC5003261

[36] F. Masayuki , Y. Tanihiro , O. Kenkichi , Y. Shozo , Biochim. Biophys. Acta Lipids Lipid Metab. 1984, 795, 458–465.

[37] D. E. Shieh , L. T. Liu , C. C. Lin , Anticancer Res. 2000, 20, 2861–2865. 11062694

[38] Z. H. Shao , T. L. Vanden Hoek , Y. Qin , L. B. Becker , P. T. Schumacker , C. Q. Li , L. Dey , E. Barth , H. Halpern , G. M. Rosen , C. S. Yuan , Am. J. Physiol. Heart Circ. Physiol. 2002, 282, 999–1006. 10.1152/ajpheart.00163.2001 11834498

[39] K. Yuki , W. Bu , R. G. Eckenhoff , T. Yokomizo , T. Okuno , Biochem. Biophys. Res. Commun. 2020, 525, 909–914. 32171526 10.1016/j.bbrc.2020.03.037 PMC7167355

[40] A. Rossi , C. Pergola , A. Koeberle , M. Hoffmann , F. Dehm , P. Bramanti , S. Cuzzocrea , O. Werz , L. Sautebin , Br. J. Pharmacol. 2010, 161, 555–570. 20880396 10.1111/j.1476-5381.2010.00930.x PMC2990155

[41] S. M. Jones , M. Luo , M. Peters-Golden , T. G. Brock , J. Biol. Chem. 2003, 278, 10257–10263. 12525477 10.1074/jbc.M211021200

[42] S. M. Jones , M. Luo , A. M. Healy , M. Peters-Golden , T. G. Brock , J. Biol. Chem. 2002, 277, 38550–38556. 12140292 10.1074/jbc.M206070200

[43] L. Zeng , Q. Zhang , G. Gerona-Navarro , N. Moshkina , M. M. Zhou , Structure 2008, 16, 643–652. 18400184 10.1016/j.str.2008.01.010 PMC3339198

[44] L. Xu , A. Cheng , M. Huang , J. Zhang , Y. Jiang , C. Wang , F. Li , H. Bao , J. Gao , N. Wang , J. Liu , J. Wu , C. C. L. Wong , K. Ruan , FEBS J. 2017, 284, 3422–3436. 28815970 10.1111/febs.14198

[45] M. Philpott , J. Yang , T. Tumber , O. Fedorov , S. Uttarkar , P. Filippakopoulos , S. Picaud , T. Keates , I. Felletar , A. Ciulli , S. Knapp , T. D. Heightman , Mol. Biosyst. 2011, 7, 2899–2908. 21804994 10.1039/c1mb05099k

[46] S. Mujtaba , L. Zeng , M. M. Zhou , Oncogene 2007, 26, 5521–5527. 17694091 10.1038/sj.onc.1210618

[47] F. Winston , C. D. Allis , Nat. Struct. Biol. 1999, 6, 601–604. 10404206 10.1038/10640

