# Supplementary material for: Identification of a Bromodomain‐like Region in 15‐Lipoxygenase‐1 Explains Its Nuclear Localization
Source: Angew Chem Int Ed Engl. 2021 Aug 31;60(40):21875–83. doi: 10.1002/anie.202106968 (PMC8518382; doi:10.1002/anie.202106968)
Supplement: Supplementary file 1 — Supporting Information [file ANIE-60-21875-s001.pdf]

Angewandte Chemie
Eine Zeitschrift der Gesellschaft Deutscher Chemiker

Supporting Information

Identification of a Bromodomain-like Region in 15-Lipoxygenase-1 Explains Its Nuclear Localization

*Deng Chen+, Zhangping Xiao+, Hao Guo, Dea Gogishvili, Rita Setroikromo, Petra E. van der Wouden, and Frank J. Dekker**

anie_202106968_sm_miscellaneous_information.pdf

Supporting Information

Table of Contents

1. Experimental Procedures
   1.1. Synthesis
   1.2. Preparation of recombinant human 15-LOX-1
   1.3. Purification of recombinant human 15-LOX-1
   1.4. 15-LOX-1 recombinant enzyme inhibition assay
   1.5. Kits-Wilson kinetic analysis
   1.6. Immunoblotting Analysis
   1.7. ABPP-based LOX inhibitor specificity validation
   1.8. Confocal microscopy
   1.9. Acetylated histone H3 peptide array
   1.10. 15-LOX-1/Histone peptide-binding ELISA
2. Supporting figures and tables
   Figure S1. Characterization of ABPs against 15-LOX-1 recombinant
   Figure S2. Uncropped western blot for Fig. 3a,b
   Figure S3. **Labelox B** labeled endogenous LOXs are immunoprecipitated by streptavidin beads.
   Figure S4. IC50 of EDTA on recombinant 15-LOX-1 crude lysate
   Figure S5. Acetylation on lysine 27 residue is essential for H3 and 15-LOX-1 binding
   Figure S6. Putative acetyl-histone binding region of 15-LOX-1 based on the theoretical model
   Table S1. The hillslope and IC_{50} of PD146176 on LOXs
   Table S2. The hillslope and IC_{50} of Baicalein on LOXs
   Table S3. The hillslope and IC_{50} of Zileuton on LOXs
3. The NMR spectra and HRMS of probes

1. Experimental Procedures

1.1. Synthesis

General. All the reagents and solvents were purchased from Sigma-Aldrich, AK Scientific, Fluorochem or Acros and were used without further purification. Reactions were monitored by thin-layer chromatography (TLC) method, in which Merck silica gel 60 F_{254} plates were used, and spots were detected with potassium permanganate staining. MP Ecochrom silica 32-63, 60 Å was used for column chromatography. Nuclear magnetic resonance spectra, ^{1}H NMR (500 MHz) and ^{13}C NMR (126 MHz), were recorded on a Bruker Avance 500 spectrometer. Chemical shifts were reported in ppm. Chemical shifts were referenced to the residual proton and carbon signals of the deuterated solvent, $CDCl_3$: δ = 7.26 ppm (^{1}H) and 77.05 ppm (^{13}C). The following abbreviations were used for spin multiplicity: s (singlet), d (doublet), t (triplet), q (quartet), dd (double of doublets), and m (multiplet). Coupling constants were reported in Hertz (Hz). High-resolution mass spectra were recorded using Fourier Transform Mass Spectrometry (FTMS) and electrospray ionization (ESI) on an Applied Biosystems/SCIEX API3000-triple quadrupole mass spectrometer.

Synthesis of probes

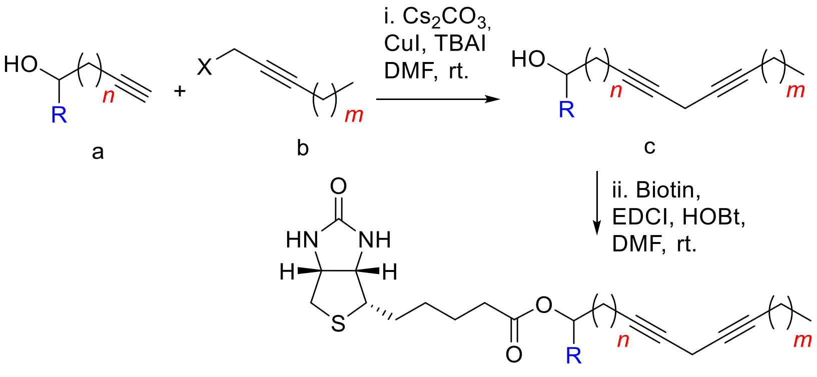

Probe	X	R	*n*	*m*
1	Br	H	0	1
2	Br	CH_3	0	1
3	Cl	H	0	4
4	Br	H	1	1
5	Br	H	2	1
6	Cl	H	2	4
7	Br	H	3	1
8	Cl	H	3	4

Scheme 1. Synthesis route of probes.

General Procedure for the synthesis of probes **1-8** (using probe **7** as an example). Synthesis of **7a**. To a mixture of CuI (728 mg, 3.8 mM), TBAI (820 mg 2.2 mM), Cs_2CO_3 (850 mg, 2.6 mM), 1-Br-2-Pentyne (206 µL, 2.0 mM) in anhydroud DMF (5.0 mL), 5-Hexyn-1-OL (220 µL, 2.0 mM) were added. The resulting yellow suspension was stirred for 20 hours at room temperature. The mixture was diluted in ethyl acetate (20 mL) and filtered through a pad of Celite. The organic solution was washed with brine (20 mL). Subsequently, the organic layer was dried over $MgSO_4$, filtered and concentrated

under reduced pressure. The product was purified using column chromatography with petroleum ether:EtOAc 20:1 (v/v) as an eluent. Product was obtained as 230 mg transparent oil-like liquid with yield of 70%. R_f value 0.5 (CH_2Cl_2/MeOH, *v/v*, 20:1). ^{1}H NMR (500 MHz, Chloroform-*d*) δ 3.66 (t, *J* = 6.4 Hz, 2H), 3.11 (p, *J* = 2.3 Hz, 2H), 2.23 – 2.14 (m, 4H), 1.69 – 1.64 (m, 2H), 1.58 (d, *J* = 8.5 Hz, 2H), 1.11 (t, *J* = 7.5 Hz, 3H). ^{13}C NMR (126 MHz, $CDCl_3$) δ 81.88, 80.06, 74.95, 73.75, 62.46, 31.85, 24.94, 18.52, 13.88, 12.39, 9.68. Synthesis of *Undeca-5,8-diyn-1-yl 5-((4S)-2-oxohexahydro-1H-thieno[3,4-d]imidazol-4-yl)pentanoate*, **7**. A mixture of EDCI (228 mg, 1.2 mmol), HOBt (162 mg, 1.2 mmol), Biotin (256 mg, 1.0 mmol), and **7a** (140 mg, 0.85 mmol) were suspended in DMF (4.0 mL) and TEA (1.0 mL). The yellow suspension was stirred at room temperature overnight and monitored with TLC. The mixture was diluted in ethyl acetate (30 mL) and washed with brine (20 mL). Subsequently, the organic layer was dried over $MgSO_4$, filtered and concentrated under reduced pressure. The product was purified using column chromatography with petroleum DCM:MeOH 30:1 (v/v) as eluent. The 93 mg of off-white solid was obtained as product and yield was 15%. R_f value 0.4 (CH_2Cl_2/MeOH, *v/v*, 10:1). ^{1}H NMR (500 MHz, $CDCl_3$) *δ* 5.62 (s, 1H), 5.32 (s, 1H), 4.51 (t, *J* = 5.5 Hz, 1H), 4.31 (t, *J* = 5.5 Hz, 1H), 4.07 (t, *J* = 6.5 Hz, 2H), 3.15 (m, 1H), 3.11 (s, 2H), 2.78 (m, 2H), 2.32 (t, *J* = 7.5 Hz, 1H), 2.19 (m, 4H), 1.70 (m, 6H), 1.55 (m, 2H), 1.45 (m, 2H), 1.11 (t, *J* = 7.5 Hz, 3H). ^{13}C NMR (126 MHz, $CDCl_3$) *δ* 173.83, 163.52, 82.05, 79.83, 75.24, 73.84, 64.13, 62.07, 60.24, 55.49, 40.69, 34.04, 28.49, 28.40, 27.95, 25.31, 24.93, 18.55, 14.02, 12.52, 9.82.

Octa-2,5-diyn-1-yl 5-((4S)-2-oxohexahydro-1H-thieno[3,4-d]imidazol-4-yl)pentanoate, **1**. Same procedure was used as synthesis of probe **7**. 1-Br-2-Pentyne (280 μL, 2.7 mmol) and 2-propyn-1-OL (157 μL, 2.7 mmol) were used as starting material to prepare the intermediate, which was subsequently coupled with Biotin (481.2 mg, 1.97 mmol) to provide the targeting product as pale yellow solid. 160 mg product was obtained with a yield 23% for two steps. R_f value 0.4 (CH_2Cl_2/MeOH, *v/v*, 10:1). ^{1}H NMR (500 MHz, $CDCl_3$) *δ* 5.74 (s, 1H), 5.25 (s, 1H), 4.70 (t, *J* = 2.5 Hz, 2H), 4.54 (t, *J* = 5.0 Hz, 1H), 4.35 (t, *J* = 4.5 Hz, 1H), 3.22 (t, *J* = 2.5 Hz, 2H), 3.18 (m, 1H), 2.80 (m, 2H), 4.20 (t, *J* = 7.5 Hz, 2H), 2.19 (qt, *J* = 7.5, 2.5 Hz, 2H), 1.74 (m, 2H), 1.47 (m, 2H), 1.14 (t, *J* = 7.5 Hz, 3H).^{13}C NMR (126 MHz, $CDCl_3$) *δ* 172.93, 163.60, 82.63, 81.91, 74.13, 72.31, 61.95, 60.17, 55.37, 52.53, 40.57, 33.63, 28.28, 28.21, 24.64, 13.83, 12.36, 9.91. HRMS calculated for $C_{18}H_{25}N_2O_3S$ $[M + H]^+$: 349.1580, found 349.1580.

Nona-3,6-diyn-2-yl 5-((4S)-2-oxohexahydro-1H-thieno[3,4-d]imidazol-4-yl)pentanoate, **2.** Probe **2** was synthesized by using the method described for **7**. 40 mg oil-like product was obtained as product with overall yield of 10%. R_f value 0.4 (CH_2Cl_2/MeOH, *v/v*, 10:1). ^{1}H NMR (500 MHz, $CDCl_3$) *δ* 5.54 (s, 1H), 5.48 (q, *J* = 4.0 Hz, 2H), 5.12 (s, 1H), 4.54 (t, *J* = 5.0 Hz, 1H), 4.35 (t, *J* = 4.5 Hz, 1H), 3.19 (s, 2H), 2.88

(m, 2H), 2.37 (m, 2H), 2.19 (*J* = 7.5, 2.0 Hz, 2H), 1.71 (m, 6H), 1.50 (d, *J* = 6.5 Hz, 3H), 1.28 (q, *J* = 7.0 Hz, 2H), 1.14 (t, *J* = 7.0 Hz, 3H). ^{13}C NMR (126 MHz, $CDCl_3$) δ 172.50, 163.30, 61.91, 60.51, 60.12, 55.36, 55.34, 40.57, 33.92, 33.89, 29.72, 28.25, 24.79, 24.69, 24.67, 21.51, 13.83, 12.38, 9.83. HRMS calculated for $C_{19}H_{27}N_2O_3S$ [M + H]$^+$: 363.1737, found 363.1738.

***Undeca-2,5-diyn-1-yl 5-((4S)-2-oxohexahydro-1H-thieno[3,4-d]imidazol-4-yl)pentanoate*, 3, D04**. Probe **3** was synthesized by using the method described for **7**. 200 mg pale yellow solid was was obtained as product with overall yield of 35%. R_f value 0.4 (CH_2Cl_2/MeOH, *v/v*, 10:1). ^{1}H NMR (500 MHz, $CDCl_3$) δ 6.16 (s, 1H), 5.55 (s, 1H), 4.55 (t, *J* = 2.0 Hz, 2H), 4.52 (t, *J* = 5.5 Hz, 1H), 4.32 (t, *J* = 5.5 Hz, 1H), 3.21 (t, *J* = 2.0 Hz, 2H), 3.17 (m, 1H), 2.84 (m, 2H), 2.39 (td, *J* = 7.5, 1.5 Hz, 2H), 2.16 (tt, *J* = 7.5, 2.5 Hz, 2H), 1.69 (m, 2H), 1.48 (m, 4H), 1.35 (m, 4H), 0.90 (t, *J* = 7.0 Hz, 1H). ^{13}C NMR (126 MHz, $CDCl_3$) δ 173.07, 163.78, 81.93, 81.38, 74.12, 72.88, 61.96, 60.14, 55.51, 52.49, 40.57, 33.71, 31.06, 28.37, 28.30, 28.22, 24.71, 22.20, 18.65, 13.99, 9.92. HRMS calculated for $C_{21}H_{31}N_2O_3S$ [M + H]$^+$: 391.2050, found 391.2051.

***Nona-3,6-diyn-1-yl 5-((3aS,4S,6aR)-2-oxohexahydro-1H-thieno[3,4-d]imidazol-4-yl)pentanoate*, 4.** Probe **4** was synthesized by using the method described for **7**. 30 mg oil-like product was obtained as product with overall yield of 38%. R_f value 0.4 (CH_2Cl_2/MeOH, *v/v*, 10:1). ^{1}H NMR (500 MHz, Chloroform-*d*) δ 4.51 (dd, *J* = 7.7, 4.8 Hz, 1H), 4.31 (dd, *J* = 7.8, 4.6 Hz, 1H), 4.13 (t, *J* = 6.9 Hz, 2H), 3.17 – 3.13 (m, 1H), 3.11 (p, *J* = 2.2 Hz, 2H), 2.91 (dd, *J* = 12.8, 5.0 Hz, 1H), 2.74 (d, *J* = 12.8 Hz, 1H), 2.52 – 2.47 (m, 2H), 2.34 (t, *J* = 7.5 Hz, 2H), 2.20 – 2.12 (m, 2H), 1.68 (td, *J* = 17.0, 14.9, 8.2 Hz, 4H), 1.50 – 1.39 (m, 2H), 1.11 (t, *J* = 7.5 Hz, 3H). ^{13}C NMR (126 MHz, $CDCl_3$) δ 173.54, 163.76, 82.22, 76.52, 76.04, 73.44, 62.50, 62.16, 60.34, 55.58, 40.66, 33.90, 28.45, 28.37, 24.86, 19.39, 13.99, 12.49, 9.83. HRMS calculated for $C_{19}H_{27}N_2O_3S$ [M + H]$^+$: 363.1742, found 363.1738.

***Deca-4,7-diyn-1-yl 5-((3aS,4S,6aR)-2-oxohexahydro-1H-thieno[3,4-d]imidazol-4-yl)pentanoate*, 5**. Probe **5** was synthesized by using the method described for **7**. 28 mg oil-like product was obtained as product with overall yield of 34%. R_f value 0.4 (CH_2Cl_2/MeOH, *v/v*, 10:1). ^{1}H NMR (500 MHz, Chloroform-*d*) δ 4.57 – 4.52 (m, 1H), 4.35 (dd, *J* = 7.6, 4.6 Hz, 1H), 4.16 (t, *J* = 6.3 Hz, 2H), 3.19 (s, 1H), 3.17 – 3.11 (m, 2H), 2.94 (d, *J* = 8.4 Hz, 1H), 2.78 (d, *J* = 12.8 Hz, 1H), 2.36 (d, *J* = 7.4 Hz, 2H), 2.32 – 2.25 (m, 2H), 2.24 – 2.15 (m, 2H), 1.84 (p, *J* = 6.7 Hz, 2H), 1.75 – 1.67 (m, 4H), 1.50 – 1.44 (m, 2H), 1.14 (t, *J* = 7.5 Hz, 3H). ^{13}C NMR (126 MHz, $CDCl_3$) δ 173.79, 173.79, 163.73, 82.10, 79.00, 75.53, 73.70, 63.29, 62.13, 60.32, 55.52, 40.66, 34.00, 28.48, 28.37, 27.89, 24.90, 15.65, 14.00, 12.51, 9.81. HRMS calculated for $C_{20}H_{29}N_2O_3S$ [M + H]$^+$: 377.1893, found 377.1892.

***Trideca-4,7-diyn-1-yl 5-((3aS,4S,6aR)-2-oxohexahydro-1H-thieno[3,4-d]imidazol-4-yl)pentanoate*, 6**. Probe **6** was synthesized by using the method described for **7**. 23 mg oil-like product

was obtained as product with overall yield of 23%. *R*$_f$ value 0.4 (CH_2Cl_2/MeOH, *v/v*, 10:1). ^{1}H NMR (500 MHz, Chloroform-*d*) δ 4.54 (s, 1H), 4.34 (s, 1H), 4.14 (t, *J* = 6.3 Hz, 2H), 3.17 (s, 1H), 3.14 – 3.05 (m, 2H), 2.93 (d, *J* = 12.4 Hz, 1H), 2.76 (d, *J* = 12.7 Hz, 1H), 2.33 (t, *J* = 7.3 Hz, 2H), 2.29 – 2.19 (m, 2H), 2.19 – 2.06 (m, 2H), 1.82 (p, *J* = 6.7 Hz, 2H), 1.74 – 1.62 (m, 4H), 1.53 – 1.38 (m, 4H), 1.36 – 1.28 (m, 4H), 0.89 (t, *J* = 7.1 Hz, 3H). ^{13}C NMR (126 MHz, $CDCl_3$) δ 173.75, 170.53, 80.88, 78.96, 75.60, 74.29, 63.33, 62.20, 60.43, 55.44, 40.66, 33.98, 31.22, 28.59, 28.47, 28.38, 27.93, 24.89, 22.35, 18.84, 15.67, 14.13, 9.86. HRMS calculated for $C_{23}H_{35}N_2O_3S$ $[M + H]^+$: 419.2363, found 419.2363.

***Hexadeca-5,8-diyn-1-yl 5-((4S)-2-oxohexahydro-1H-thieno[3,4-d]imidazol-4-yl)pentanoate*, 8**. Probe **8** was synthesized by using the method described for **7**. 24 mg oil-like product was obtained as product with overall yield of 12%. *R*$_f$ value 0.4 (CH_2Cl_2/MeOH, *v/v*, 10:1). ^{1}H NMR (500 MHz, $CDCl_3$) *δ* 5.20 (s, 1H), 4.90 (s, 1H), 4.55 (t, *J* = 6.5 Hz, 1H), 4.35 (t, *J* = 6.5 Hz, 1H), 4.11 (t, *J* = 6.5 Hz, 2H), 3.19 (m, 1H), 3.15 (m, 2H), 2.85 (m, 2H), 2.36 (t, *J* = 7.5 Hz, 2H), 2.24 (tt, *J* = 7.0, 2.5 Hz, 2H), 2.18 (tt, *J* = 7.0, 2.5 Hz, 2H), 1.79-1.67 (m, 6H), 1.61-1.31 (m, 10H), 0.92 (t, *J* = 7.0 Hz, 3H). ^{13}C NMR (126 MHz, $CDCl_3$) *δ* 173.70, 163.41, 80.68, 79.67, 75.17, 74.30, 64.02, 61.95, 60.14, 55.33, 40.57, 33.89, 31.09, 28.46, 28.36, 28.27, 27.81, 25.19, 24.78, 22.23, 18.71, 18.43, 14.01, 9.73.

1. Synthesis of control probes

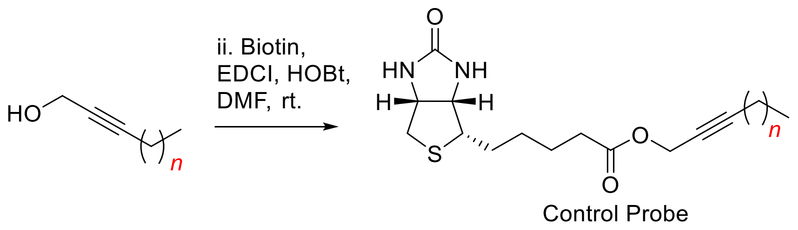

Control Probe	*n*
**9**	0
**10**	2

Scheme 2. Synthesis route of control probes.

***Prop-2-yn-1-yl 5-((4S)-2-oxohexahydro-1H-thieno[3,4-d]imidazol-4-yl)pentanoate*, 9**. A mixture of EDCl (134 mg, 0.7 mmol), HOBt (95mg, 0.7 mmol), Biotin (147 mg, 0.6 mmol), and 2-Propyn-1-OL (28.87 µl, 0.5 mmol) were suspended in DMF (4.0 mL) and TEA (1.0 mL). The yellow suspension was stirred overnight at room temperature. The mixture was diluted in water and filtered through a pad of Celite and the organic layer was washed with ethyl acetate (30 mL) and brine. Subsequently, the organic layer was dried over $MgSO_4$, filtered and concentrated under reduced pressure. The product was purified using column chromatography with petroleum DCM:MeOH 20:1 (v/v) as an eluent. *R*$_f$ value 0.3 (CH_2Cl_2/MeOH, *v/v*, 10:1). The yield was 46%. ^{1}H NMR (500 MHz, $CDCl_3$) *δ* 6.24 (s, 1H), 5.75 (s, 1H), 5.31 (s, 1H), 4.68 (s, 2H), 4.51 (t, *J* = 7.5 Hz, 1H), 4.31 (t, *J* = 7.5 Hz, 1H), 3.16 (qt, *J* = 7.0, 1.5 Hz, 2H), 2.90 (dd, *J* = 13.0, 5.0 Hz, 2H), 2.76 (d, *J* = 13.0 Hz, 1H), 2.50 (t, *J* = 3.0 Hz,

1H), 2.40 (t, *J* = 7.5 Hz, 2H), 1.77-1.64 (m, 4H), 1.47 (m, 2H). ^{13}C NMR (126 MHz, $CDCl_3$) δ 172.88, 163.96, 77.80, 74.94, 61.97, 60.14, 55.53, 51.88, 40.59, 33.62, 28.31, 28.22, 24.66. HRMS calculated for $C_{13}H_{18}N_2O_3S$ $[M + H]^+$: 283.1111, found 283.1112.

***Hex-2-yn-1-yl 5-((4S)-2-oxohexahydro-1H-thieno[3,4-d]imidazol-4-yl)pentanoate*, 10.** A mixture of EDCl (134mg, 0.7 mmol), HOBt (95mg, 0.7 mmol), Biotin (147mg 0.6 mmol), and 2-Propyn-1-OL (55 µL, 0.5 mM) were suspended in DMF (2.0 mL) and TEA (0.5 mL). The suspension was stirred overnight at room temperature. The mixture was diluted in water and filtered through a pad of Celite and the organic layer was washed with ethyl acetate (30 mL) and brine. Subsequently, the organic layer was dried over $MgSO_4$, filtered and concentrated under reduced pressure. The product was purified using column chromatography with DCM:MeOH 20:1 (v/v) as an eluent. The yield was 62%. R_f value 0.3 (CH_2Cl_2/MeOH, *v/v*, 10:1). ^{1}H NMR (500 MHz, $CDCl_3$) δ 5.35 (s, 1H), 4.92 (s, 1H), 4.54 (tt, *J* = 5.0, 1.0 Hz, 2H), 4.35 (tt, *J* = 5.0, 1.0 Hz, 1H), 3.19 (m, 1H), 2.96 (dd, *J* = 13.0, 5.0 Hz, 1H), 2.77 (d, *J* = 13.0 Hz, 1H), 2.41 (d, *J* = 7.5 Hz, 2H), 2.22 (tt, *J* = 7.0, 2.0 Hz, 2H), 1.79-1.67 (m, 4H), 1.61-1.43 (m, 4H), 1.00 (t, *J* = 7.5 Hz, 3H). ^{13}C NMR (126 MHz, $CDCl_3$) δ 173.05, 164.18, 87.46, 74.15, 61.97, 60.15, 55.58, 52.71, 40.54, 33.70, 28.32, 28.19, 24.67, 21.83, 20.69, 13.46. HRMS calculated for $C_{16}H_{25}N_2O_3S$ $[M + H]^+$: 325.1580, found 325.1580.

1.2. Preparation of recombinant human 15-LOX-1

The 15-LOX-1 protein was produced in *E. coli* BL21(DE3) as described elsewhere[1]. Briefly, a single colony was used to inoculate 2YT medium with 50 µg/ml kanamycin (Duchefa, Haarlem, The Netherlands). After overnight culturing at 37°C, 200 rpm, 10 ml of such culture was used to inoculate 1 L 2YT medium with 0.2% glycerol. The cells were allowed to grow at 37°C, 200 rpm for around 1.5-2.5 h to reach 0.6 of OD600. The culture was equilibrated to 20°C, followed by adding kanamycin and IPTG to a final concentration of 50 µg/ml and 0.1 mM, respectively. The 15-LOX-1 was produced at 20°C for overnight with 200 rpm shaking. Subsequently, cells were pelleted through centrifugation at 3750 g for 20 min, after which the pellet (around 5 grams) was resuspended in 10 ml LOX assay buffer (25 mM HEPES , pH 7.5) supplemented with 1X protease inhibitor cocktail (PIC, Roche, Mannheim, Germany). Later, cells were disrupted by sonication at 50% duty cycle, 70% output for 5 cycles (45 seconds sonicating and 3 minutes cooling down for each cycle) on an ice water bath. Cell debris was pelleted through centrifugation at 4°C, 20000 g for 1 h. The aliquot of supernatant containing 15-LOX-1 enzyme was snap-frozen in liquid nitrogen and stored at -80°C for further use. BL21(DE3) transformed with

empty plasmid was proceed with the same procedure, which lysate was used as the negative control (referred as BL21 control later) in 15-LOX-1 recombinant enzyme inhibition studies.

1.3. Purification of recombinant human 15-LOX-1

The 15-LOX-1 with N-terminal his-tag was produced as described above. After removing the cell debris, the supernatant containing 15-LOX-1 was loaded on a Ni-NTA column (Qiagen, Hilden, Germany) pre-equilibrated with LOX lysis buffer. The column was washed twice with 30 column volume of LOX lysis buffer, and once with 30 column volume of wash buffer (25 mM HEPES, 300 mM NaCl, 25 mM Imidazole, pH 8.0). Later, the 15-LOX-1 was eluted twice with 2 column volume of elution buffer (25 mM HEPES, 300 mM NaCl, 250 mM Imidazole, pH 8.0), followed by desalting with a PD-10 column (Sigma-Aldrich) with LOX storage buffer (25 mM HEPES, 20% Glycerol, pH 7.5). The desalted 15-LOX-1 was further purified and concentrated with Vivaspin Turbo 15 (30.000 MWCO). The protein concentration was determined by Nanodrop spectrophotometry. The 100 µl aliquots of 15-LOX-1 were stored at -20 for further experiments.

1.4. 15-LOX-1 recombinant enzyme inhibition assay

The 15-LOX-1 inhibition assay has been detailed previously[2]. Briefly, the conversion of linoleic acid to hydroperoxy-(9Z,11E)-octadecadienoic acid (λ_{max} of 234 nm) was used to determine the activity of the 15-LOX-1 enzyme. The conversion rate was the slope of UV-absorbance at 234 nm overtime at the linear increase period.

To determine the IC_{50} of probes, the cell lysate was pre-diluted in LOX assay buffer at a ratio of 1:160. The inhibition assay was done in an F-bottom UV-STAR 96-well plate, in which 50 µl of the diluted enzyme was incubated with 140 µl of a probe diluted in LOX assay buffer for 10 min at room temperature (RT). Later 10 µl of 500 µM linoleic acid dissolved in ethanol was added to the plate to reach a final concentration of 25 µM. The UV-absorbance at 234 nm was determined by a BioTek Synergy H1 plate reader (BioTek, Winooski, VT, USA). The slope of the 15-LOX-1 sample untreated with the probe was set as 100%, while the slope of the BL21 control sample was set as 0%.

For the determination of enzyme kinetics, a series concentration of linoleic acid was used. Fifty µl of pre-diluted 15-LOX-1 enzyme was incubated with 140 µl of probe solution at a fixed concentration for 10 min at RT. Subsequently, 10 µl of a series concentration of linoleic acid was added to a final concentration ranging from 10 µM to 100 µM. The conversion rate was calculated as the same as

described in the IC_{50} determination assay. Michaelis-Menten plots, Lineweaver-Burk plots, K_m, and V_{max}, are generated using GraphPad Prism 8.3.0.

1.5. Kits-Wilson kinetic analysis

The probes inhibit the 15-LOX-1 enzyme with a two-step reaction. First, a probe binds to an enzyme to get a reversible complex, E*I. The dissociation constant of inhibitor (Ki) is from the formation of E*I. Then, the probe forms a covalent bond with the enzyme, which generates an irreversible complex, E-I. The inactivation rate constant (ki) is to describe the rate of E-I generation. To determine these inactivation parameters, Kitz-Wilson analysis was employed. The procedure has been detailed previously by our group[3]. Following the protocol, the ki and Ki of all probes with IC_{50} values less than 50 µM were determined.

1.6. Immunoblotting Analysis

Raw 264.7 cells were seeded in a 6-well plate at a density of $5x10^5$ cells per well in high glucose DMEM media supplemented with 1% penicillin/streptomycin, 10% fetal bovine serum (FBS) (Costar Europe, Badhoevedrop, The Netherlands) at 37°C and cultured overnight. Afterward, cells were treated with the desired concentration of **Labelox B** at the expected time. Then, the cells were harvested and lysed with RIPA buffer containing 1X PIC. The insoluble cell debris was removed by centrifugation. Later, 30 µl of each sample was used for immunoblotting analysis. The membrane was blocked with 5 % skimmed milk (w/v) in PBST and incubated with HRP conjugated streptavidin (150 ng/ml) overnight at 4°C. After washing, the blotting bands were detected with enhanced chemiluminescence (ECL) solution (GE Healthcare, Amersham, UK).

1.7. ABPP-based LOX inhibitor specificity validation

A549 cells were lysed by RIPA buffer supplemented with 1x PIC. After sonication and centrifugation, the supernatant was collected, and the protein concentration was determined by a Pierce BCA Protein Assay Kit (Thermo Fisher Scientific, USA). The lysates were then diluted with PBS to 300 ng/µl and stored in -20°C. To label endogenous LOXs with **Labelox B** under the competition condition, 180 µl diluted lysates were incubated with 200 µl different LOX inhibitor solution (in PBS) with desired concentrations for 18 min to inhibit LOXs activity. Later, 20 µl of a 20 µM **Labelox B** (in PBS) was added and incubated shortly for 2 min, followed by adding 50 µl of a 0.5 M EDTA to stop the labeling reaction. The reaction mixture was 450 µl in total. Inhibitors and **Labelox B** were absent in positive and

negative control, respectively. To detect the competition labeling of **Labelox B** among different LOXs, a high-binding 96-well plate (Greiner, #M4561) was coated with 5-LOX (#ab169755, Abcam), 12-LOX (#ab211506, Abcam), 15-LOX-1 (#ab80221, Abcam), and 15-LOX-2 (#ab23691, Abcam) antibody (1:500 dilution in PBS) for overnight at 4°C. After washing triply with 0.05%PBST, the plate was blocked with 2% (w/v) BSA in PBST. Subsequently, 100 µl of each reaction mixture was added to the LOX-antibody-coated wells. There are four LOX antibodies used for coating. Therefore, in total, 400 µl mixture is needed for each reaction. After one hour of incubation, LOXs were pulled down by antibodies accordingly. Next, 100 µl of HRP-conjugated streptavidin (1:2000 dilution in PBS, Thermo Fisher Scientific, USA) was added to each well to detect the D04 labeled LOXs among precipitated LOXs. After washing with PBST, 100 µl of mixed HRP substrate reagent (R&D Systems, Minneapolis, USA) was added, and the colorizing reaction was stopped by adding 100 µl of 1M sulfuric acid. The absorbance at 450 nM was determined via a Synergy H1 plate reader.

1.8. Confocal microscopy

To visualize the intracellular location of LOXs, immunofluorescence staining was applied to cells. A549 or RAW264.7 macrophage cells were seeded on glass coverslips in a 6-well plate at a density of 1x10^5 cells per well. After overnight incubation, cells were treated with 1 µg/ml LPS for 24 h. Subsequently, cells were fixed with 100% methanol (pre-cooled in -20ºC) for 5 min in a freezer. After triple washes with PBS to remove methanol, cells were treated with 50 µM **Labelox B** for 1 h at 37°C. Then, unbound probes were removed and cells were washed with PBS three times, 5 min for each. After incubating with blocking buffer (1% BSA, 22.52 mg/ml glycine in 0.1%PBST) for 1 h, cells were stained with desired LOX antibodies (1 µg/ml) or Histone antibodies (1 µg/ml) for 1 h at RT. Subsequently, cells were treated with Alexa Fluor Plus 555 conjugated goat anti-rabbit secondary antibody (1:1000 dilution, #A32732, Invitrogen, Waltham, MA. USA) and Cy2-conjugated streptavidin (1:1000 dilution, Leinco Technologies, St. Louis, USA) simultaneously. Next, coverslips were mounted onto slides with anti-fading mountant with NucBlue™ stain (Invitrogen, Waltham, MA. USA). The pictures were acquired using a Leica SP8 confocal laser scanning microscope and analyzed by ImageJ.

1.9. Acetylated histone H3 peptide array

The array kit was purchased from Abcam (ab233494), and the assay was performed following the manufacturer's instructions.

1.10. 15-LOX-1/Histone peptide-binding ELISA

Fifty µl of purified 15-LOX-1 at a concentration of 100 nM was coated on a high-binding 96-well plate. After blocking with 2% PBSTB, 50 ul of biotinylated-peptides (Anaspec, Fremont, USA) (in 0.05% PBST) with desired concentrations were applied on the plate followed by incubating at RT for 1 h. After washing triply with 0.05% PBST, 50 ul of HRP-conjugated streptavidin (500 ng/ml) was added to each well to detect the histone peptides associated with 15-LOX-1. Later, 100 µl of mixed HRP substrate reagent was added, and the colorizing reaction was stopped by adding 100 µl of 1M sulfuric acid. The absorbance at 450 nM was determined via a Synergy H1 plate reader.

2. Supporting figures and tables

A

Probe	Conc. (μM)	K_m^{app} (μM)	V_{max}^{app} (absorbance/s)
N.A.	0	17.55 ± 4.86	$14.06 \times 10^{-3} \pm 1.25 \times 10^{-3}$
1	25	22.61 ± 3.69	$11.60 \times 10^{-3} \pm 0.59 \times 10^{-3}$
	50	32.92 ± 6.87	$9.76 \times 10^{-3} \pm 0.77 \times 10^{-3}$
	100	44.99 ± 12.25	$9.02 \times 10^{-3} \pm 1.01 \times 10^{-3}$
3	0.5	10.84 ± 3.45	$10.08 \times 10^{-3} \pm 0.78 \times 10^{-3}$
	1	18.94 ± 5.33	$8.53 \times 10^{-3} \pm 0.79 \times 10^{-3}$
	2	25.08 ± 10.91	$7.01 \times 10^{-3} \pm 0.79 \times 10^{-3}$
7	7.5	17.65 ± 3.08	$11.92 \times 10^{-3} \pm 0.59 \times 10^{-3}$
	15	19.05 ± 4.90	$11.67 \times 10^{-3} \pm 0.88 \times 10^{-3}$
	30	20.10 ± 7.57	$9.87 \times 10^{-3} \pm 1.14 \times 10^{-3}$
8	12.5	14.63 ± 7.58	$10.03 \times 10^{-3} \pm 1.14 \times 10^{-3}$
	25	13.98 ± 9.60	$7.75 \times 10^{-3} \pm 1.19 \times 10^{-3}$

B

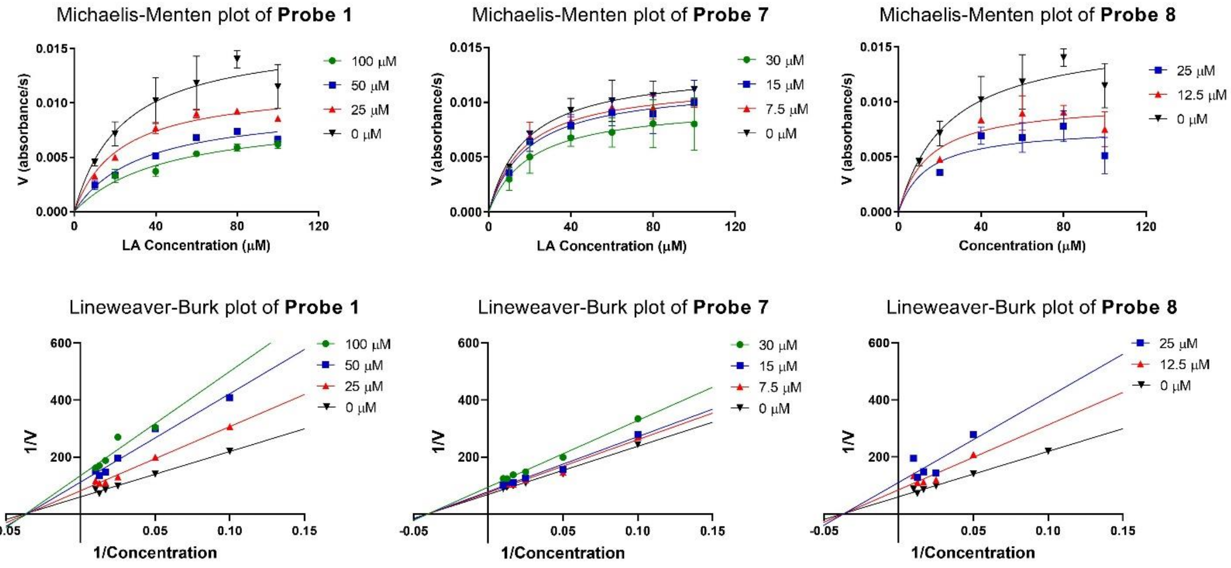

Figure S1. Characterization of ABPs against 15-LOX-1 recombinant. **A**, K_m and V_{max} of ABPs. **B**, Michaelis-Menten plots of ABPs.

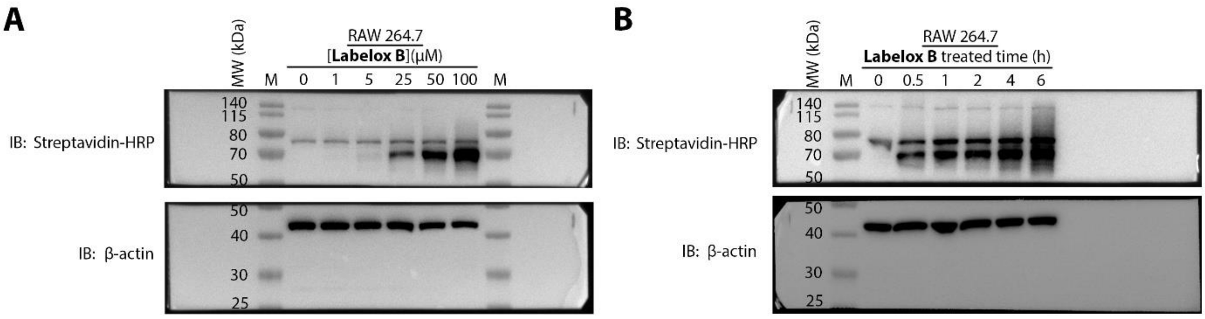

Figure S2. Uncropped western blot for Fig. 3a,b.

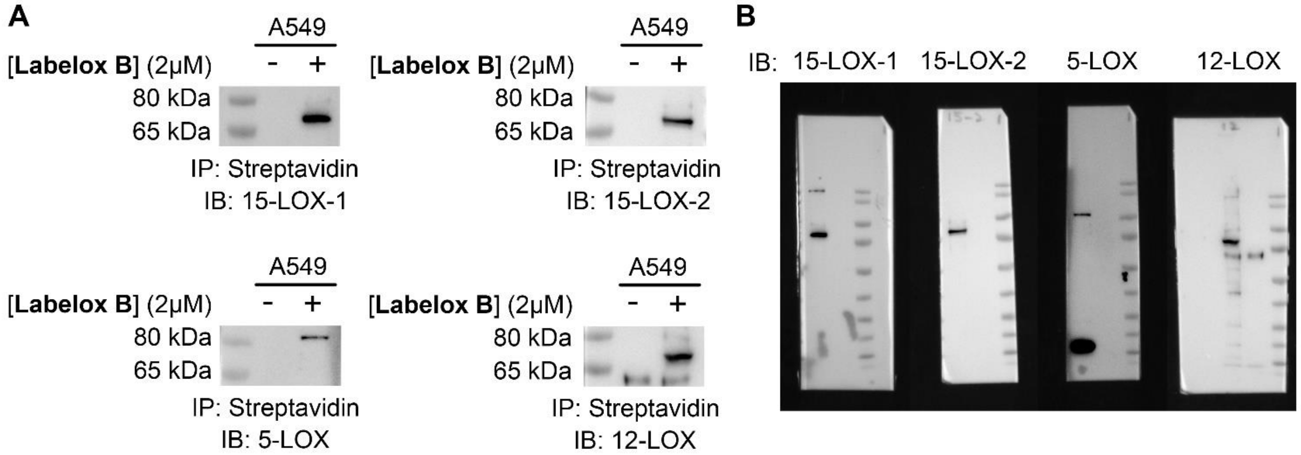

Figure S3. **Labelox B** labeled endogenous LOXs are immunoprecipitated by streptavidin beads. (A) A549 cell lysates were treated with. **Labelox B**. After labeling, free **Labelox B** was removed by Vivaspin 2. **Labelox B** labeled proteins were precipitated by Dynabeads and analyzed by Western blotting. (B) Uncropped Western blots for (A)

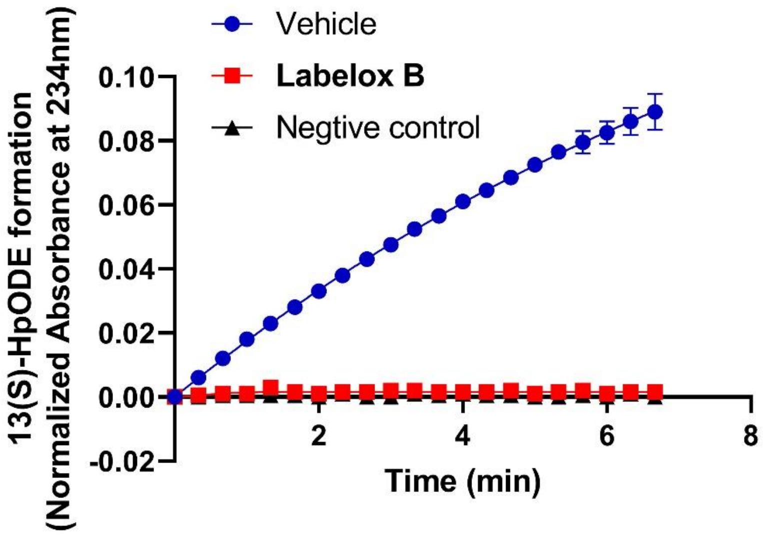

Figure S4. Jump dilution assay shows **Labelox B** irreversibly inhibits recombinant 15-LOX-1. 15-LOX-1 was incubated with **Labelox B** at 10 μM for 20min at room temperature to ensure 100% inhibition. Then, the enzyme/probe mixture was dilution 100-fold and employed for linoleic acid conversion. The group incubated with vehicle was condisered as positive control, while the group in presence of 15-LOX-1 was negative control.

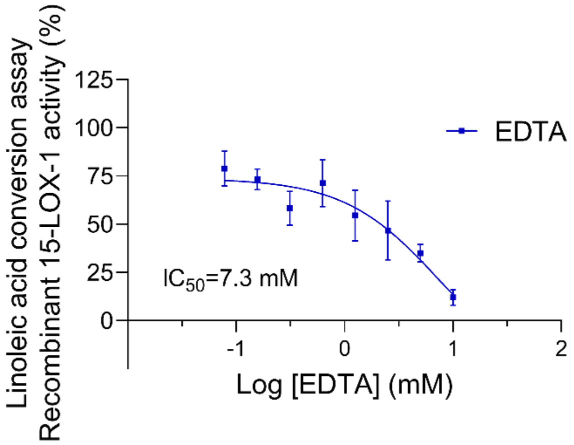

Figure S5. IC_{50} of EDTA on recombinant 15-LOX-1 crude lysate. Data are mean±s.d., n⩾2 independent experiments

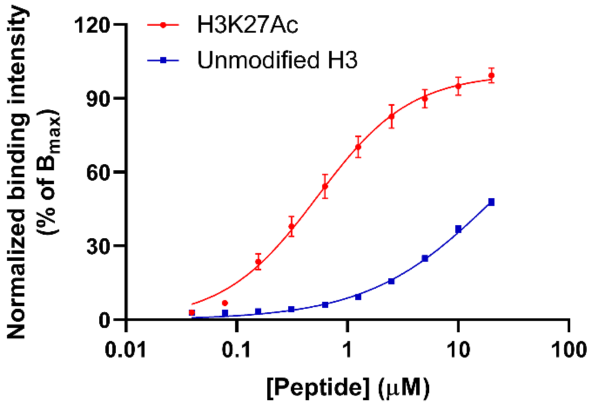

Figure S6. Acetylation on lysine 27 residue is essential for H3 and 15-LOX-1 binding. Data are mean± SME, n⩾2 independent experiments

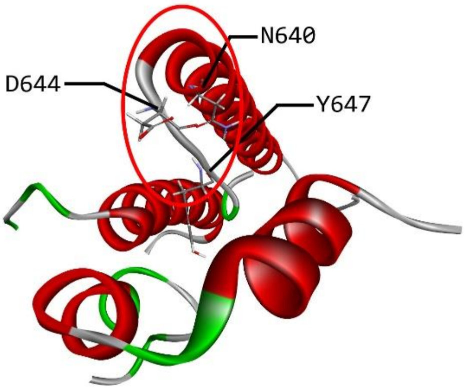

Figure S7. Putative acetyl-histone binding region of 15-LOX-1 based on the theoretical model[4]

Table S1. The hillslope and IC_{50} of PD146176 on LOXs

	PD146176					
	HillSlope			IC_{50} (μM)		
	Value	+Error	-Error	Value	+Error	-Error
5-LOX	-0.99	0.05	0.05	14.75	0.85	0.80

12-LOX	-0.98	0.05	0.06	19.12	1.19	1.11
15-LOX-1	-0.93	0.05	0.05	17.33	1.04	0.98
15-LOX-2	-0.88	0.08	0.08	122.80	16.52	12.89

Table S2. The hillslope and IC_{50} of Baicalein on LOXs

Baicalein						
	HillSlope			IC_{50} (µM)		
	Value	+Error	-Error	Value	+Error	-Error
5-LOX	-0.26	0.03	0.03	560.54	359.01	187.42
12-LOX	-0.41	0.03	0.03	273.49	76.74	53.13
15-LOX-1	-0.47	0.03	0.03	295.52	56.23	43.05
15-LOX-2	-0.34	0.03	0.03	851.85	410.69	242.99

Table S3. The hillslope and IC_{50} of Zileuton on LOXs

Zileuton						
	HillSlope			IC_{50} (µM)		
	Value	+Error	-Error	Value	+Error	-Error
5-LOX	-0.48	0.04	0.04	383.33	103.78	72.74
12-LOX	-0.47	0.11	0.15	554.12	925.86	272.77
15-LOX-1	N/A	N/A	N/A	N/A	N/A	N/A
15-LOX-2	N/A	N/A	N/A	N/A	N/A	N/A

N/A: Not applicable

References

[1] N. Eleftheriadis, S. Thee, J. Te Biesebeek, P. Van Der Wouden, B. J. Baas, F. J. Dekker, *Eur. J. Med. Chem.* **2015**, *94*, 265–275.

[2] N. Eleftheriadis, C. G. Neochoritis, N. G. J. Leus, P. E. Van Der Wouden, A. Dömling, F. J. Dekker, *J. Med. Chem.* **2015**, *58*, 7850–7862.

[3] a) N. Eleftheriadis, S. A. Thee, M. R. H. Zwinderman, N. G. J. Leus, F. J. Dekker, Angew. Chem. Int. Ed. **2016**, 55, 12300-12305; b) N. Eleftheriadis, S. A. Thee, M. R. H. Zwinderman, N. G. J. Leus, F. J. Dekker, Angew. Chem. **2016**, 128, 12488–12493.

[4] P. P. M. P.ARUL PRAKASAM, “ma-c7glw | ModelArchive,” DOI 10.5452/ma-c7glwcan be found under https://www.modelarchive.org/doi/10.5452/ma-c7glw, **2005**.

3. The NMR spectra and HRMS of probes

Probe **1**

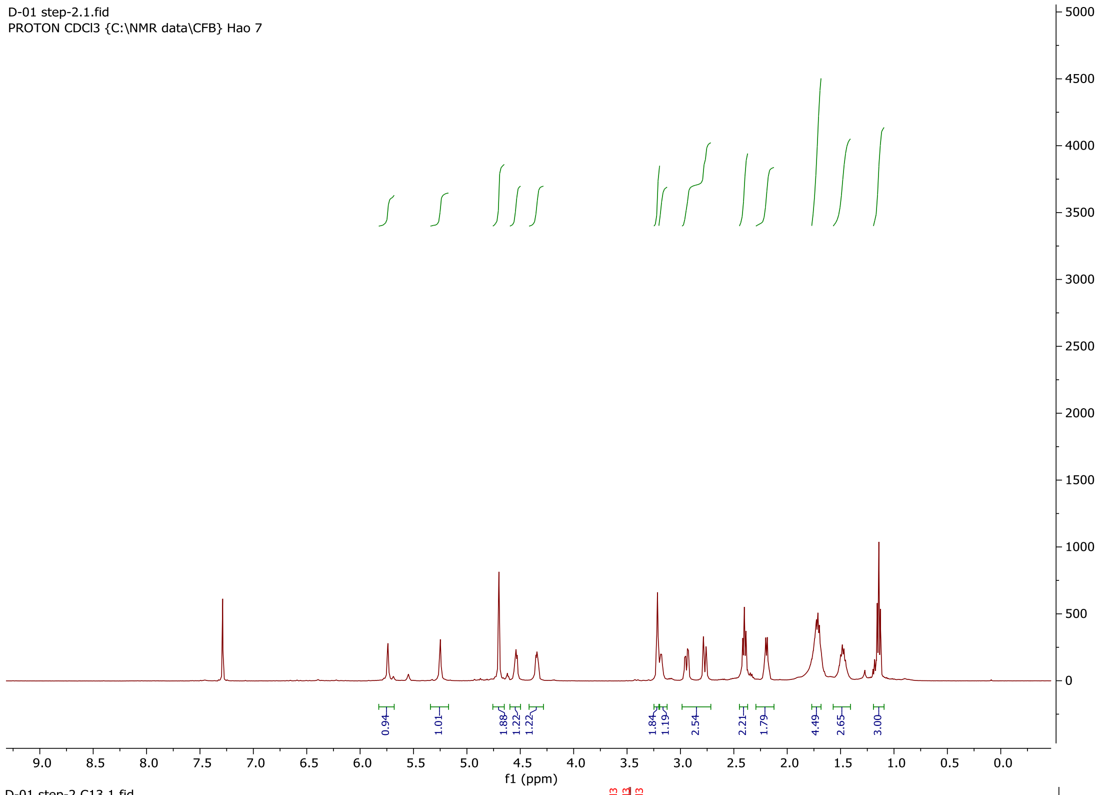

4.

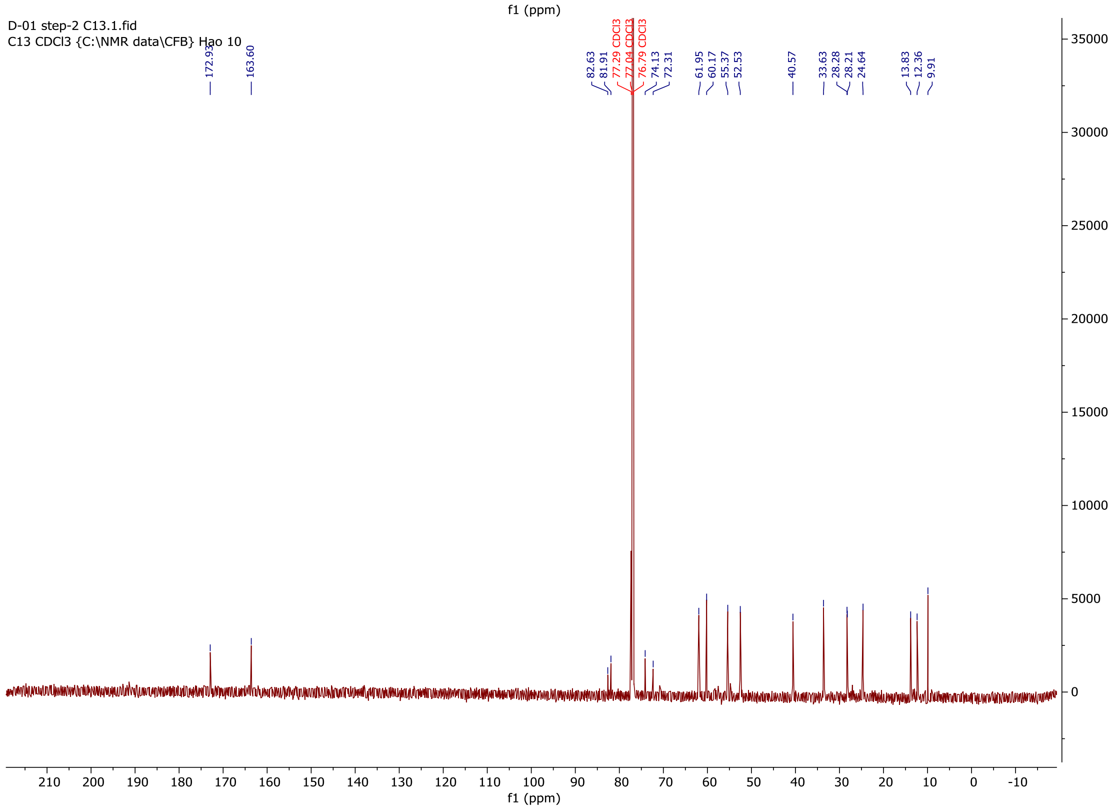

5.

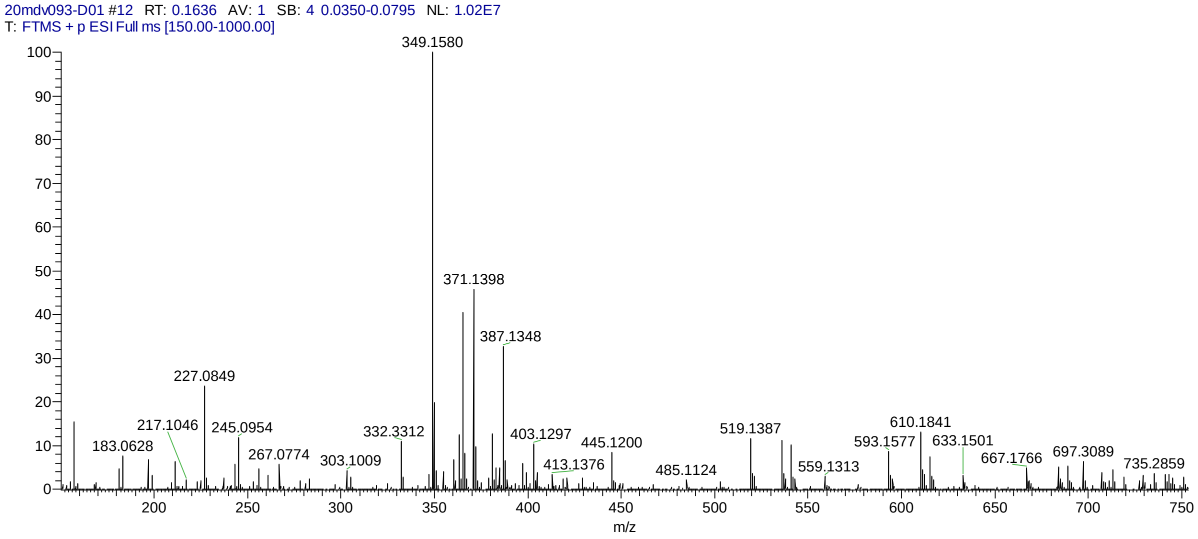
20mdv093-D01 #12 RT: 0.1636 AV: 1 SB: 4 0.0350-0.0795 NL: 1.02E7
T: FTMS + p ESI Full ms [150.00-1000.00]
349.1580
371.1398
387.1348
227.0849
217.1046
245.0954
183.0628
267.0774
303.1009
332.3312
403.1297
413.1376
445.1200
485.1124
519.1387
559.1313
593.1577
610.1841
633.1501
667.1766
697.3089
735.2859
m/z

Probe **2**

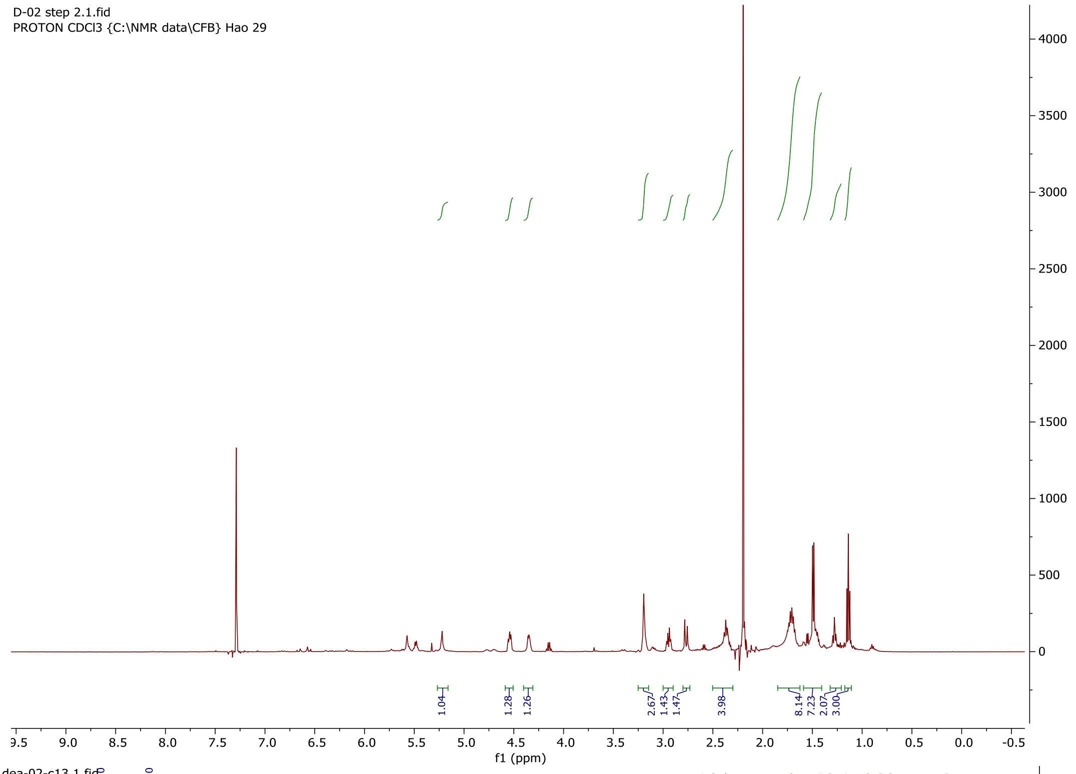
D-02 step 2.1.fid
PROTON CDCl3 {C:\NMR data\CFB} Hao 29
f1 (ppm)

6.

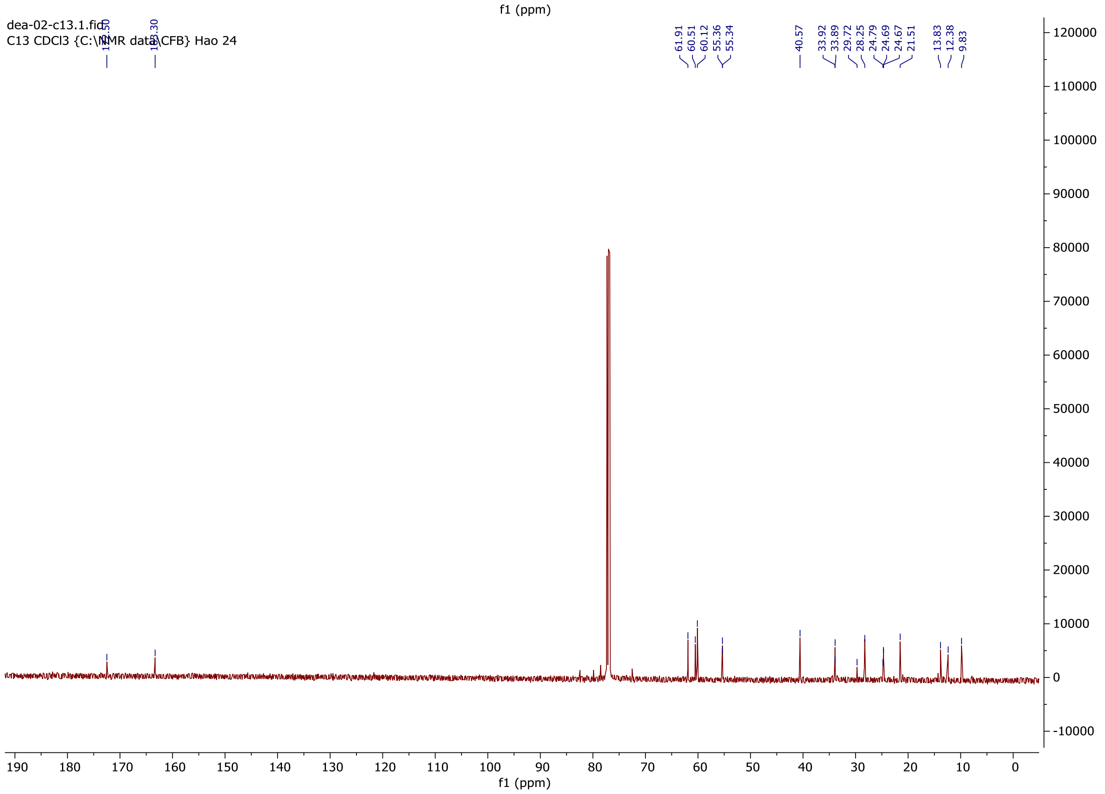
dea-02-c13.1.fid
C13 CDCl3 {C:\NMR data\CFB} Hao 24
61.91
60.51
60.12
55.36
55.34
40.57
33.92
33.89
29.72
28.25
24.79
24.69
24.67
21.51
13.83
12.38
9.83
f1 (ppm)

7.

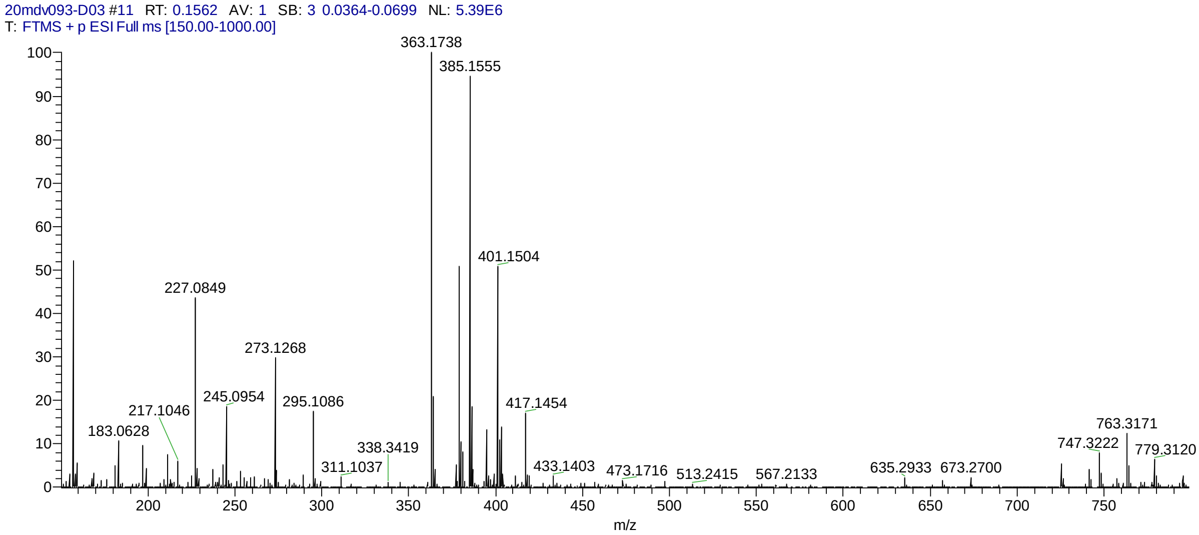
20mdv093-D03 #11 RT: 0.1562 AV: 1 SB: 3 0.0364-0.0699 NL: 5.39E6
T: FTMS + p ESI Full ms [150.00-1000.00]
363.1738
385.1555
401.1504
227.0849
273.1268
245.0954
295.1086
217.1046
183.0628
417.1454
338.3419
311.1037
433.1403
473.1716
513.2415
567.2133
635.2933
673.2700
747.3222
763.3171
779.3120
m/z

8.

9.

Probe **3**

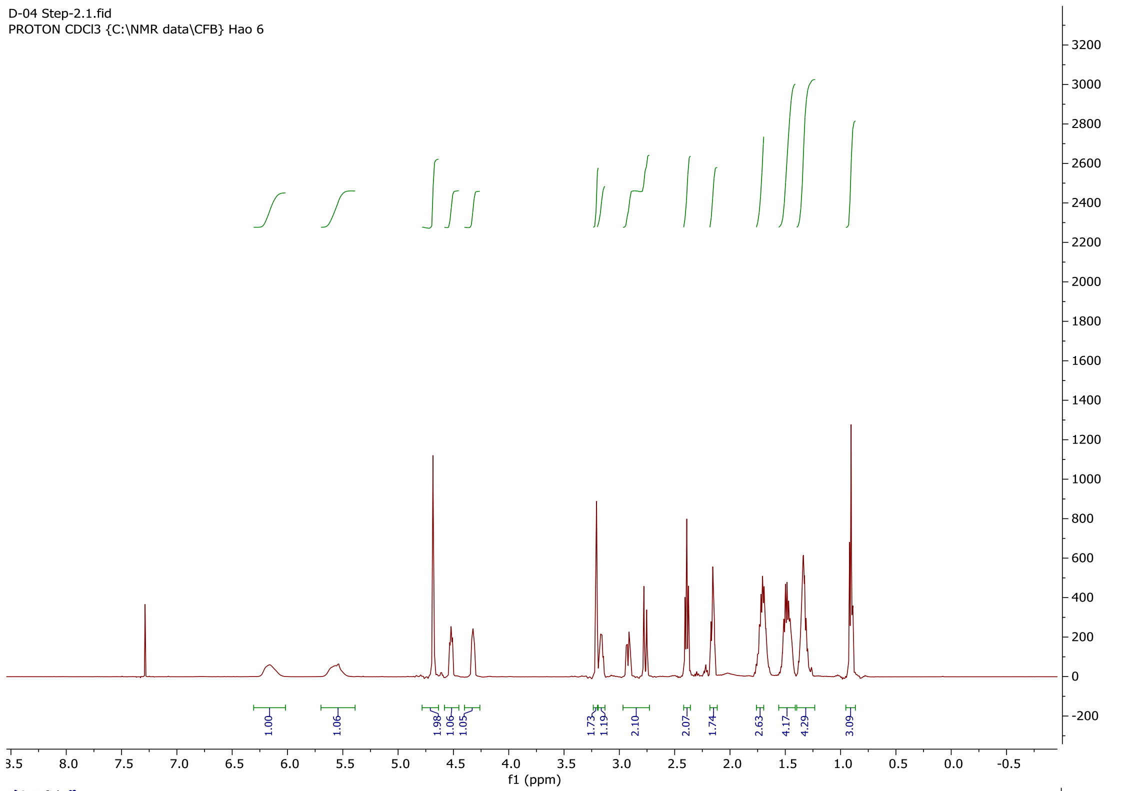
D-04 Step-2.1.fid
PROTON CDCl3 {C:\NMR data\CFB} Hao 6
1.00
1.06
1.98
1.06
1.05
1.73
1.19
2.10
2.07
1.74
2.63
4.17
4.29
3.09
f1 (ppm)

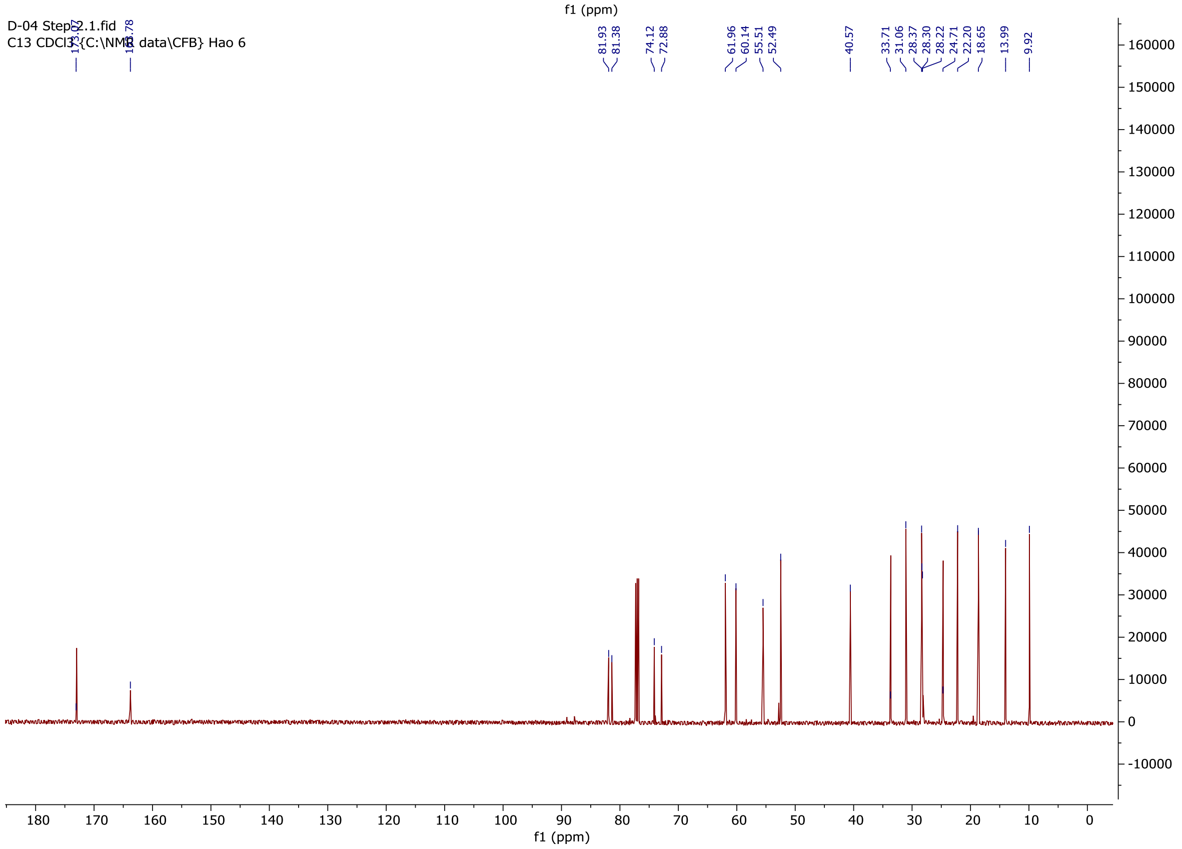
D-04 Step-2.1.fid
C13 CDCl3 {C:\NMR data\CFB} Hao 6
81.93
81.38
74.12
72.88
61.96
60.14
55.51
52.49
40.57
33.71
31.06
28.37
28.30
28.22
24.71
22.20
18.65
13.99
9.92
f1 (ppm)

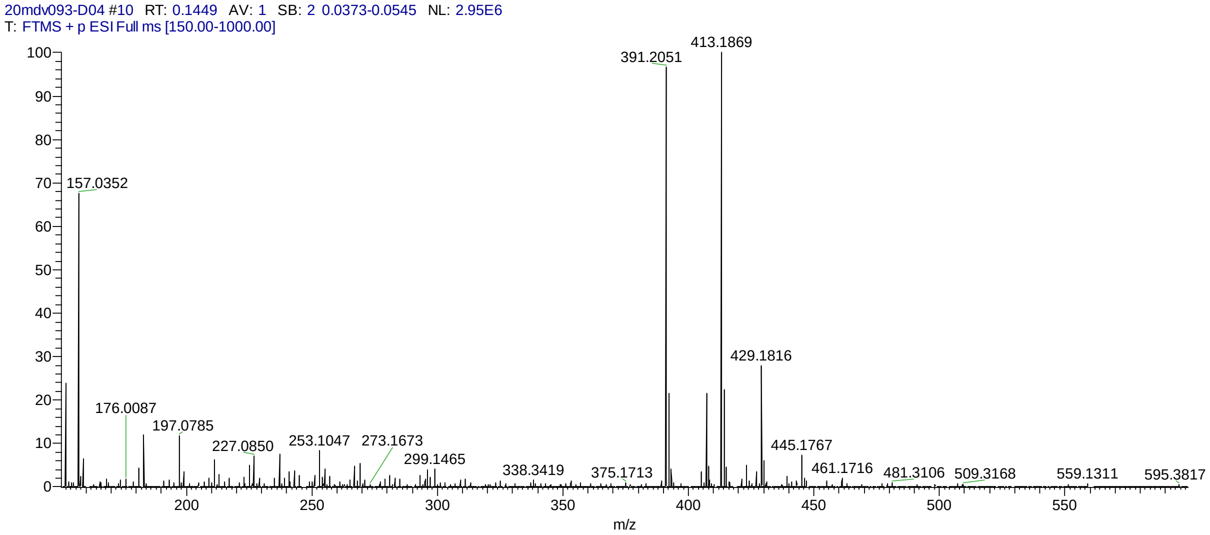
20mdv093-D04 #10 RT: 0.1449 AV: 1 SB: 2 0.0373-0.0545 NL: 2.95E6
T: FTMS + p ESI Full ms [150.00-1000.00]
413.1869
391.2051
157.0352
429.1816
176.0087
197.0785
227.0850
253.1047
273.1673
299.1465
338.3419
375.1713
445.1767
461.1716
481.3106
509.3168
559.1311
595.3817
m/z

Probe **4**

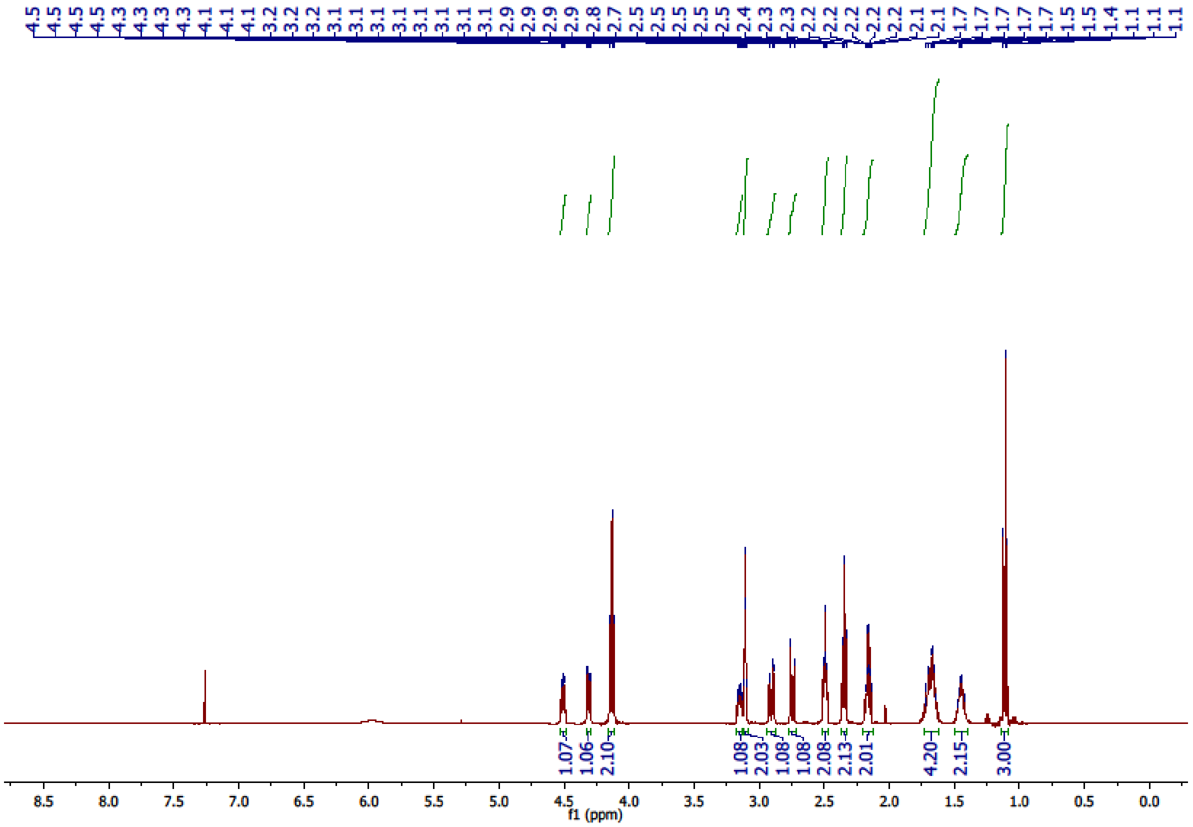
1.07
1.06
2.10
1.08
2.03
1.08
1.08
2.08
2.13
2.01
4.20
2.15
3.00
f1 (ppm)

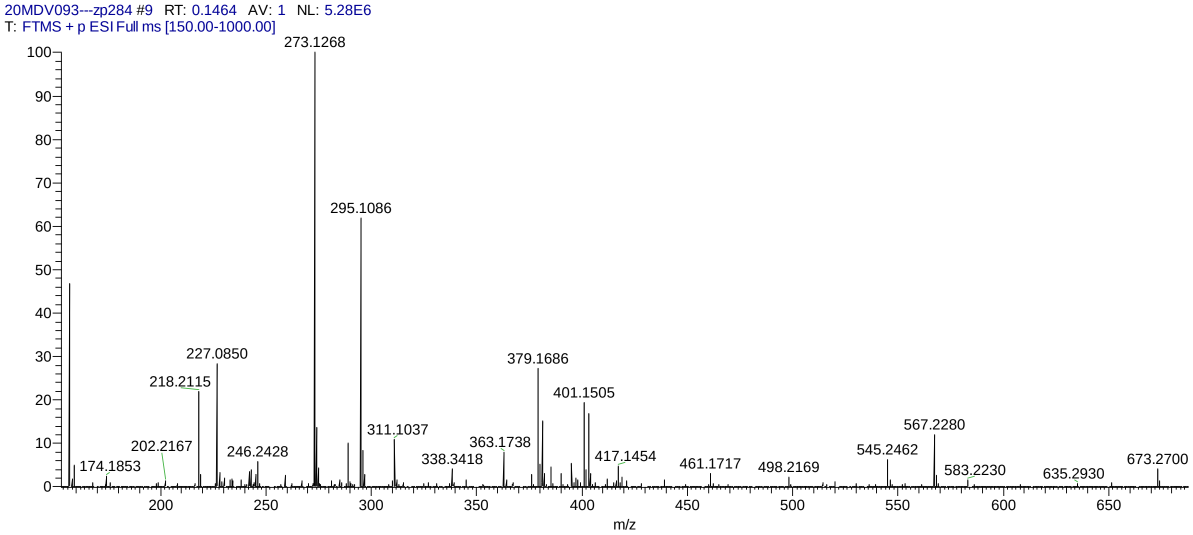
20MDV093---zp284 #9 RT: 0.1464 AV: 1 NL: 5.28E6
T: FTMS + p ESI Full ms [150.00-1000.00]
273.1268
295.1086
227.0850
218.2115
202.2167
174.1853
246.2428
311.1037
338.3418
363.1738
379.1686
401.1505
417.1454
461.1717
498.2169
545.2462
567.2280
583.2230
635.2930
673.2700
m/z

Probe **5**

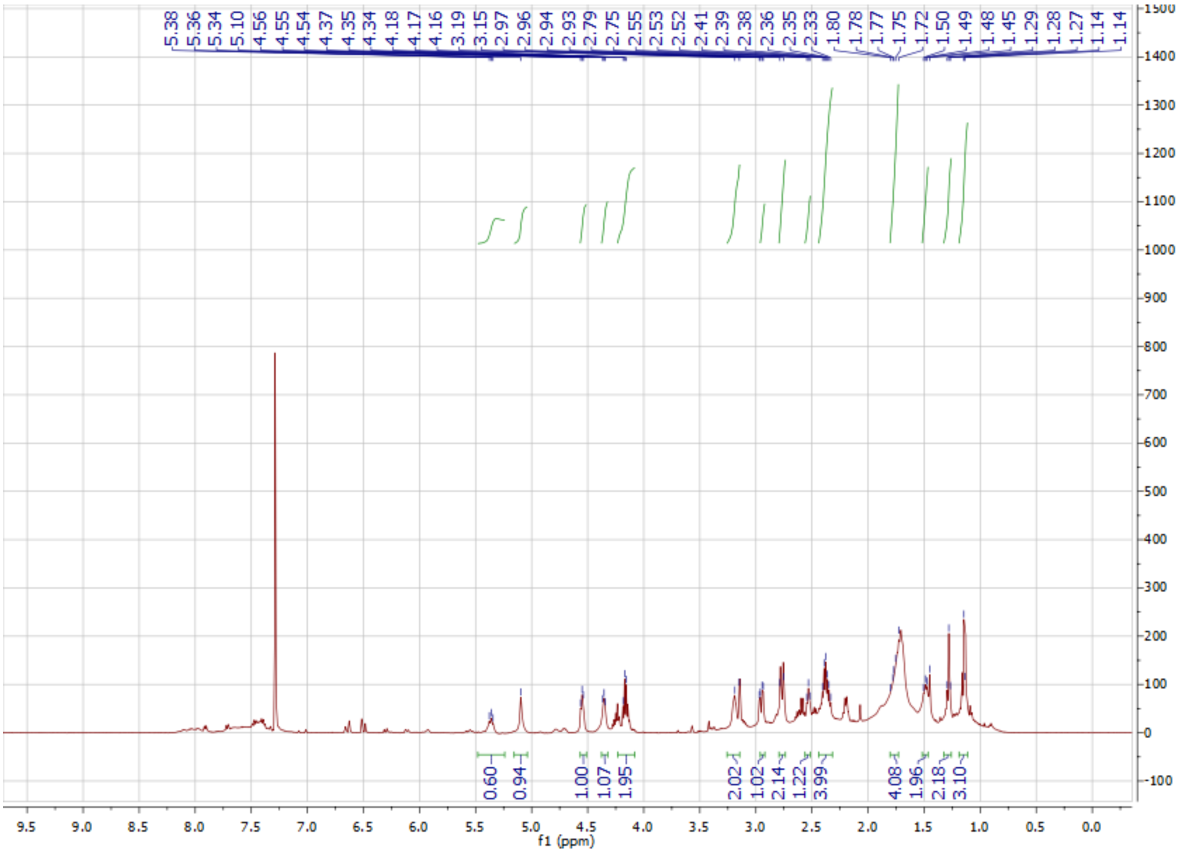

10.

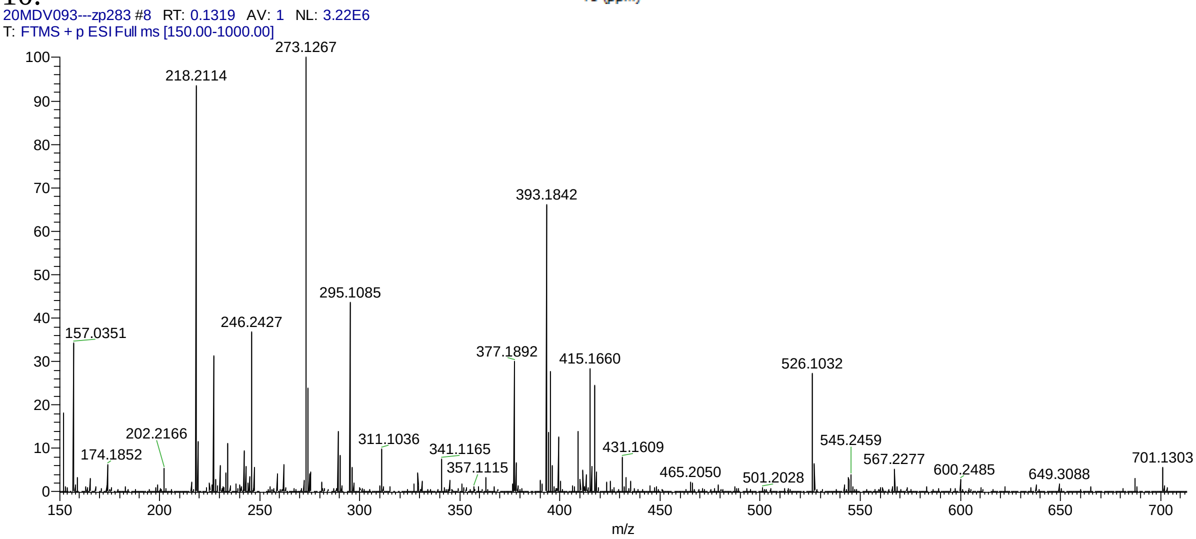
20MDV093---zp283 #8 RT: 0.1319 AV: 1 NL: 3.22E6
T: FTMS + p ESI Full ms [150.00-1000.00]
273.1267
218.2114
393.1842
295.1085
246.2427
157.0351
377.1892
415.1660
526.1032
202.2166
174.1852
311.1036
341.1165
357.1115
431.1609
545.2459
567.2277
465.2050
501.2028
600.2485
649.3088
701.1303
m/z

Probe **6**

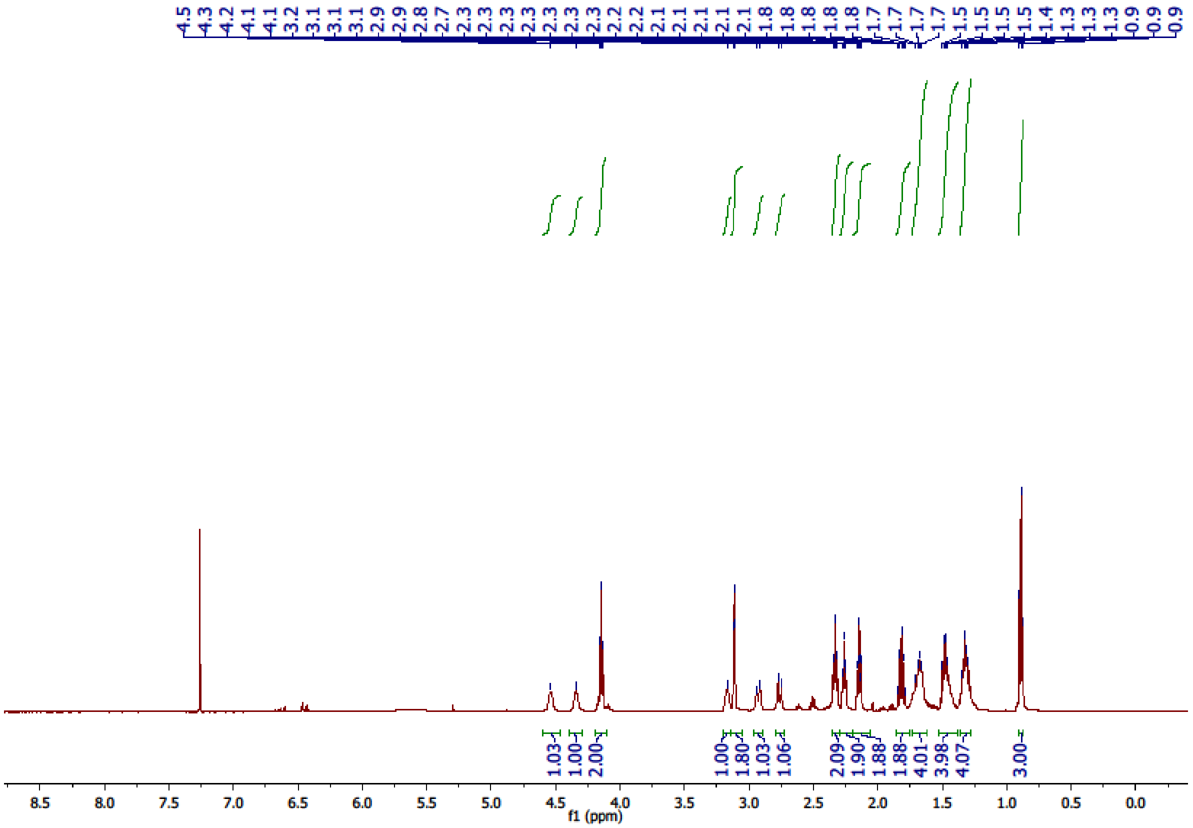

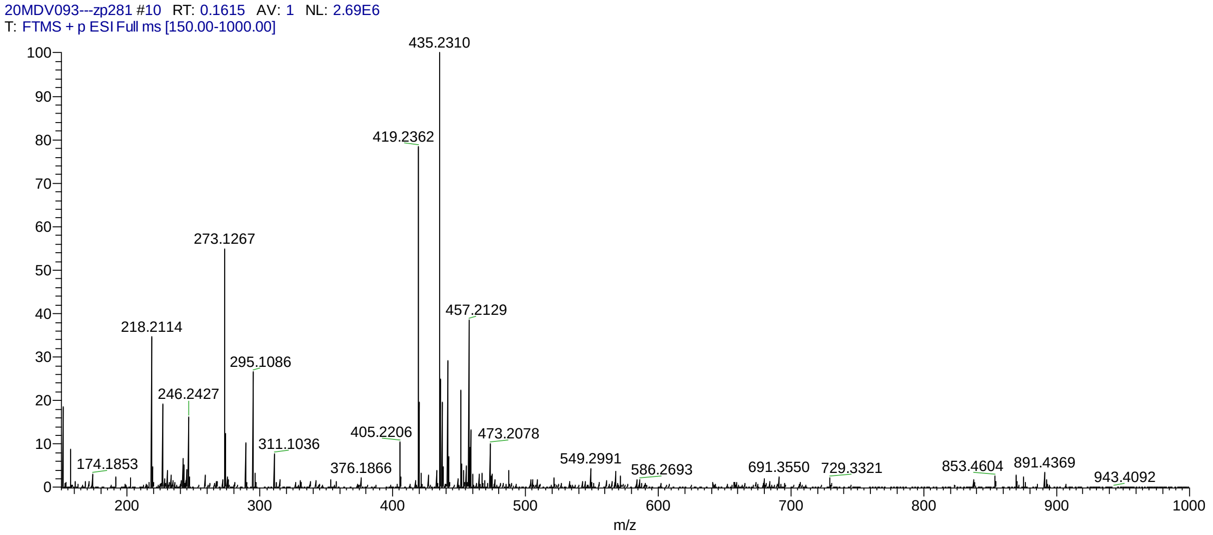
20MDV093---zp281 #10 RT: 0.1615 AV: 1 NL: 2.69E6
T: FTMS + p ESI Full ms [150.00-1000.00]
435.2310
419.2362
273.1267
457.2129
218.2114
295.1086
246.2427
311.1036
405.2206
473.2078
174.1853
376.1866
549.2991
586.2693
691.3550
729.3321
853.4604
891.4369
943.4092
m/z

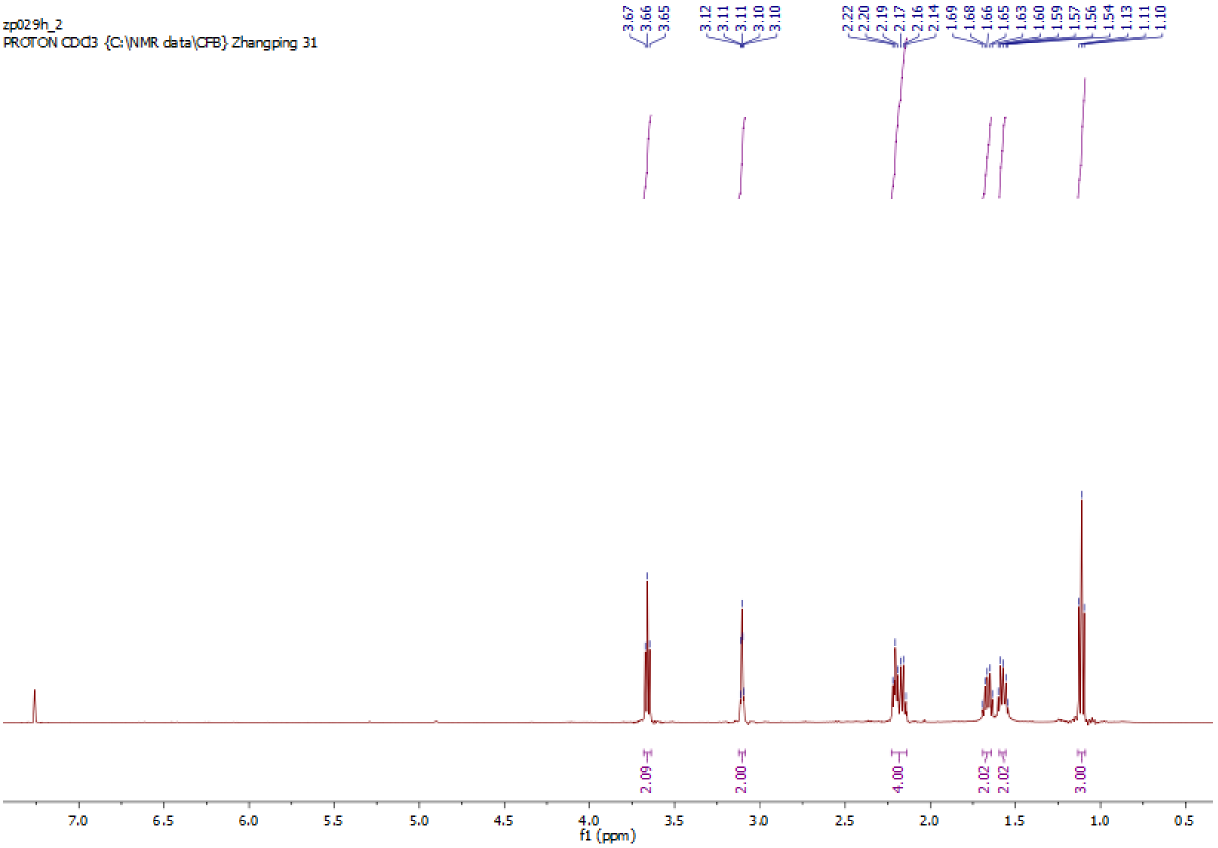
zp029h_2
PROTON CDCl3 {C:\NMR data\CFB} Zhangping 31
3.67
3.66
3.65
3.12
3.11
3.11
3.10
3.10
2.22
2.20
2.19
2.17
2.16
2.14
1.69
1.68
1.66
1.65
1.63
1.60
1.59
1.57
1.56
1.54
1.13
1.11
1.10
2.09
2.00
4.00
2.02
2.02
3.00
7.0 6.5 6.0 5.5 5.0 4.5 4.0 3.5 3.0 2.5 2.0 1.5 1.0 0.5
f1 (ppm)

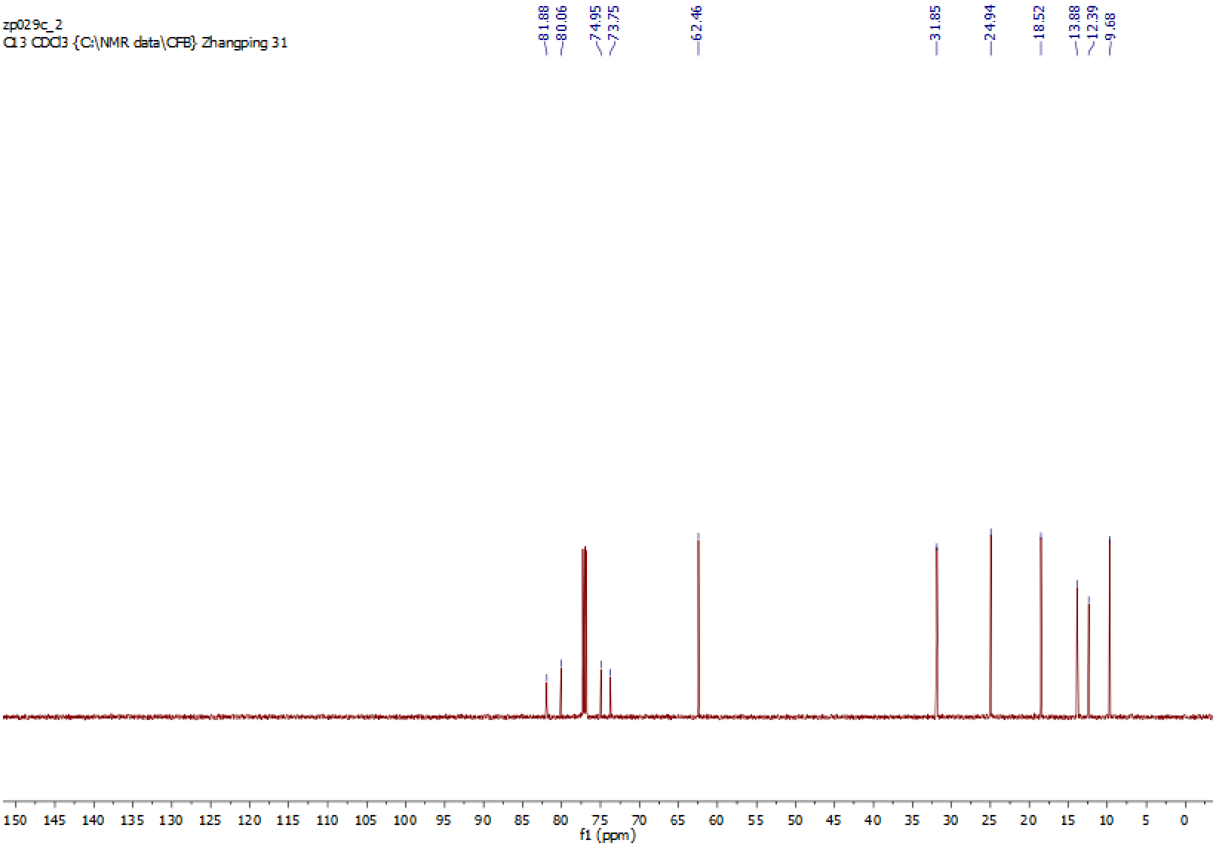
zp029c_2
C13 CDCl3 {C:\NMR data\CFB} Zhangping 31
81.88
80.06
74.95
73.75
62.46
31.85
24.94
18.52
13.88
12.39
9.68
150 145 140 135 130 125 120 115 110 105 100 95 90 85 80 75 70 65 60 55 50 45 40 35 30 25 20 15 10 5 0
f1 (ppm)

Probe **7**

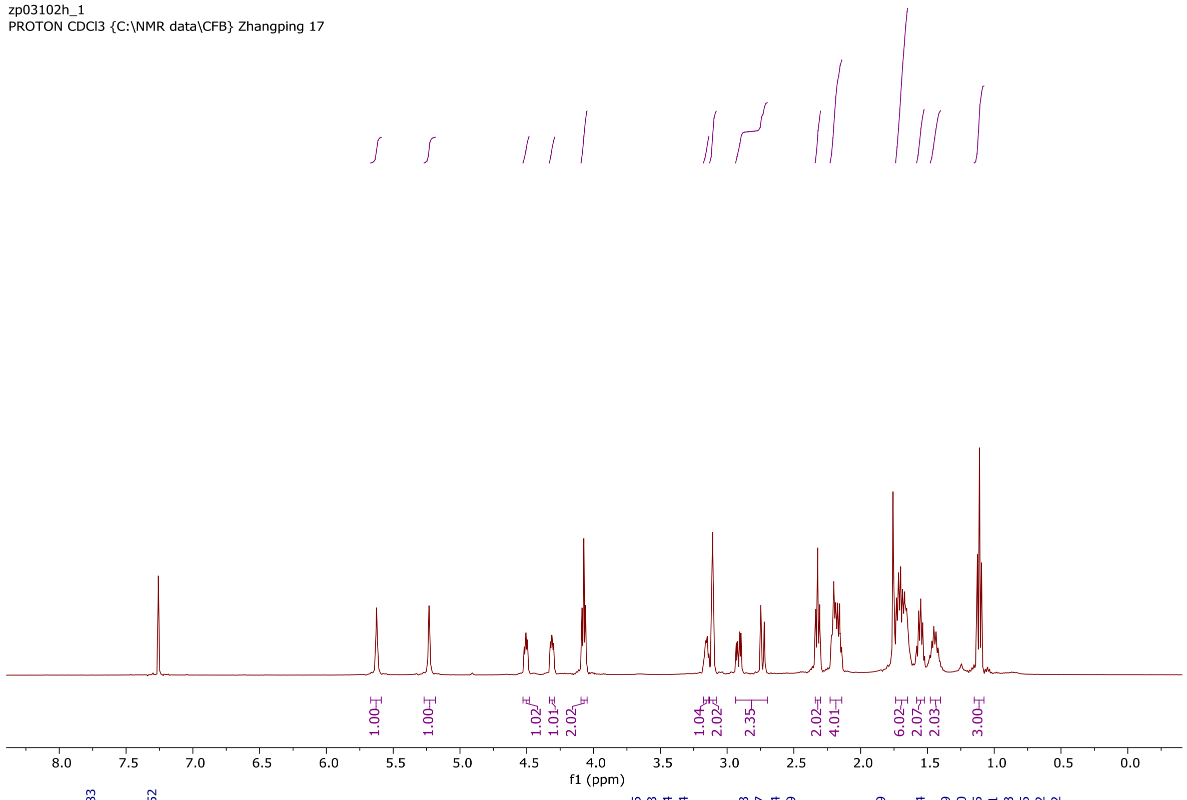
zp03102h_1
PROTON CDCl3 {C:\NMR data\CFB} Zhangping 17
1.00
1.00
1.02
1.01
2.02
1.04
2.02
2.35
2.02
4.01
6.02
2.07
2.03
3.00
8.0
7.5
7.0
6.5
6.0
5.5
5.0
4.5
4.0
3.5
3.0
2.5
2.0
1.5
1.0
0.5
0.0
f1 (ppm)

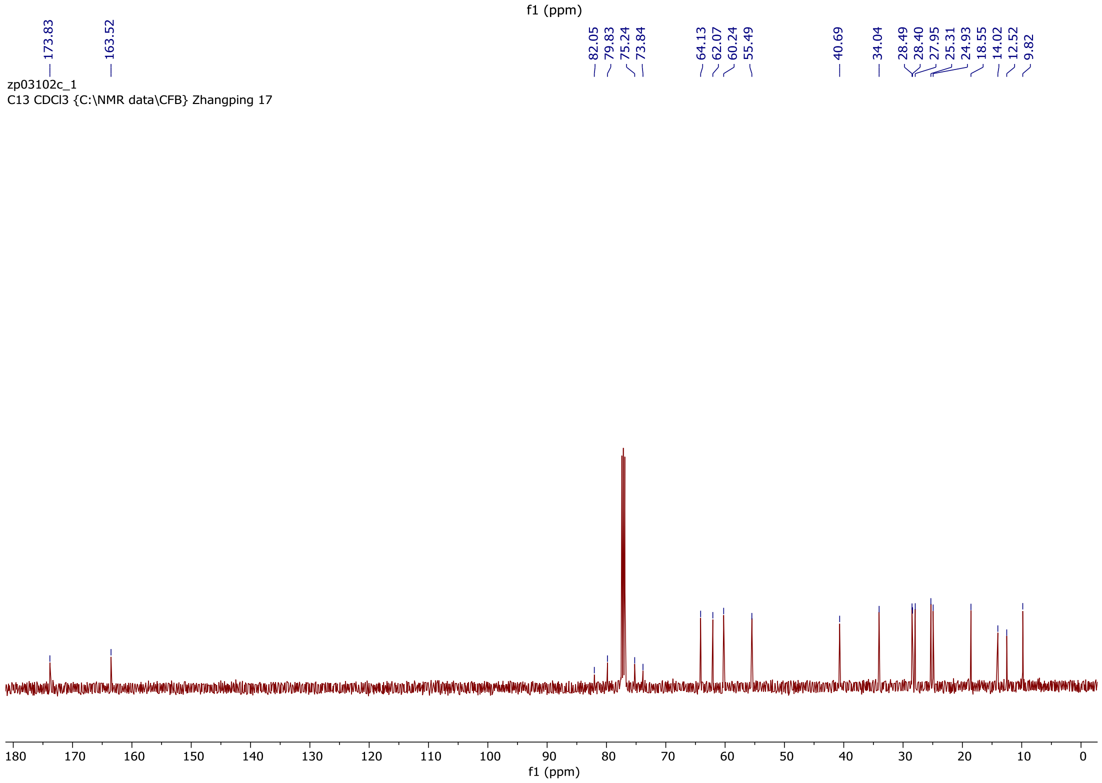
173.83
163.52
82.05
79.83
75.24
73.84
64.13
62.07
60.24
55.49
40.69
34.04
28.49
28.40
27.95
25.31
24.93
18.55
14.02
12.52
9.82
zp03102c_1
C13 CDCl3 {C:\NMR data\CFB} Zhangping 17
180
170
160
150
140
130
120
110
100
90
80
70
60
50
40
30
20
10
0
f1 (ppm)

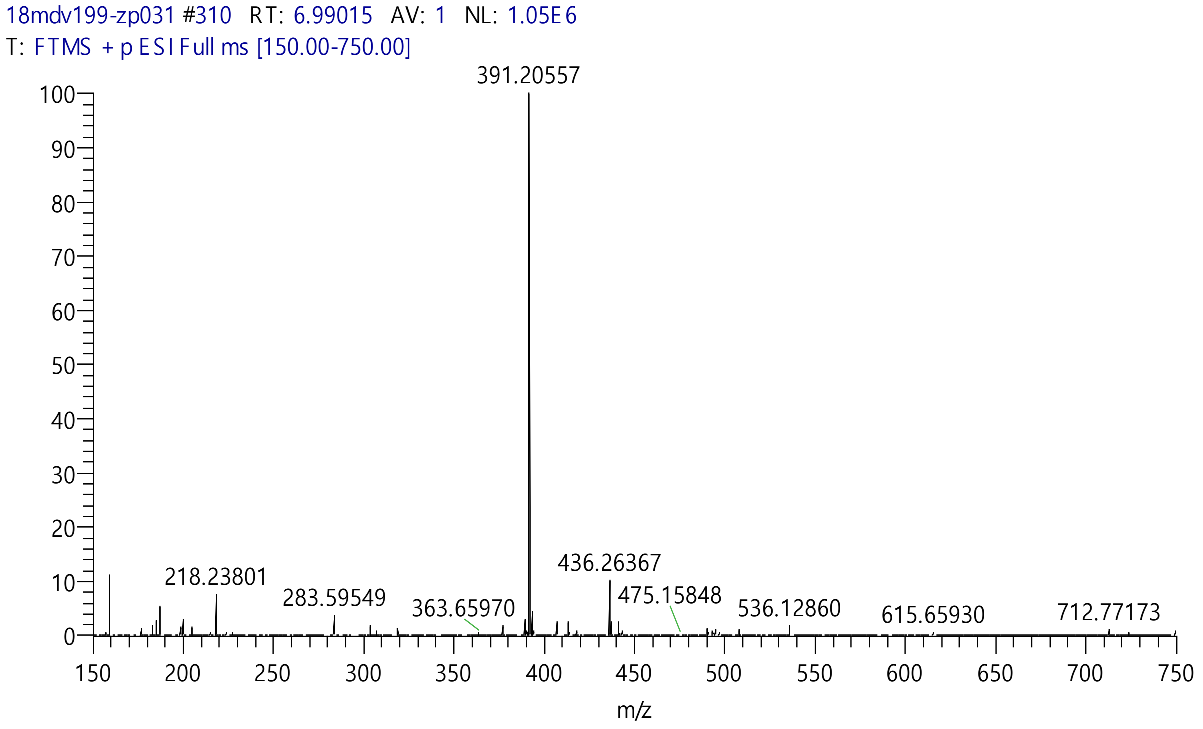
18mdv199-zp031 #310 RT: 6.99015 AV: 1 NL: 1.05E6
T: FTMS + p ESI Full ms [150.00-750.00]
391.20557
218.23801
283.59549
363.65970
436.26367
475.15848
536.12860
615.65930
712.77173
m/z

Probe **8**

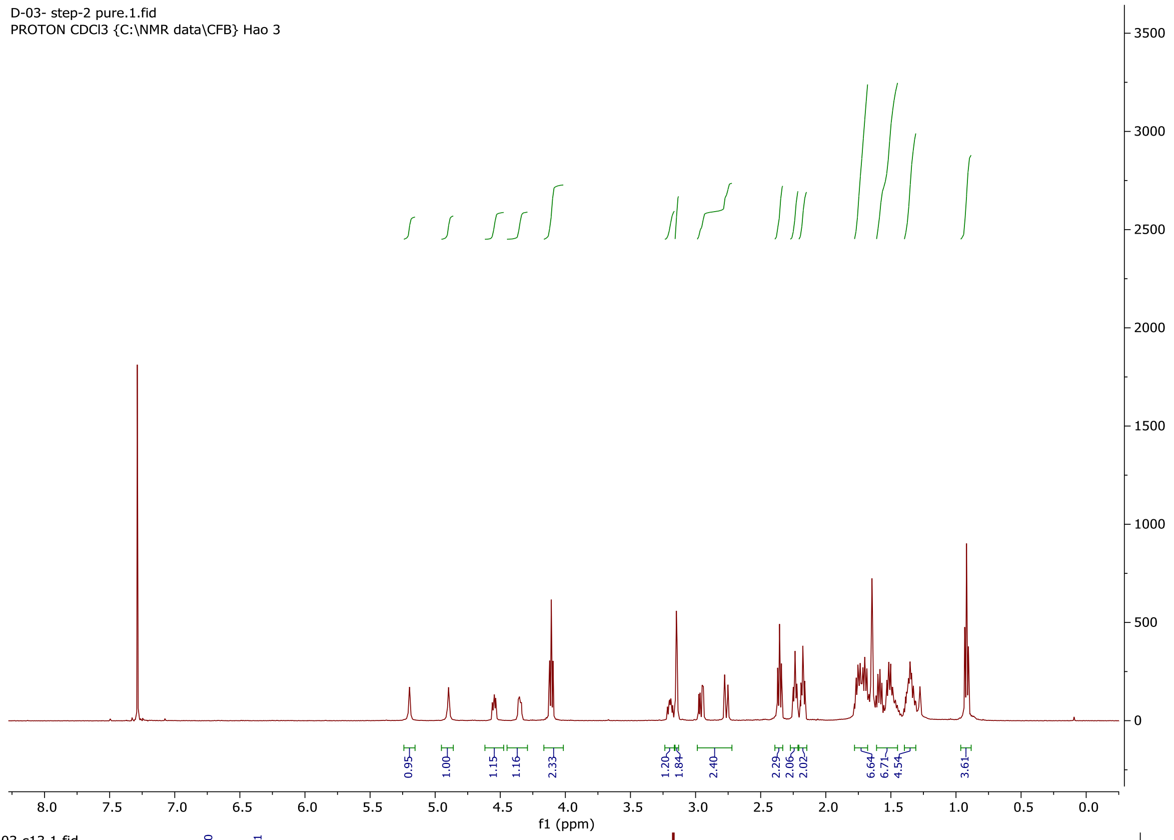
D-03- step-2 pure.1.fid
PROTON CDCl3 {C:\NMR data\CFB} Hao 3
0.95
1.00
1.15
1.16
2.33
1.20
1.84
2.40
2.29
2.06
2.02
6.64
6.71
4.54
3.61
f1 (ppm)

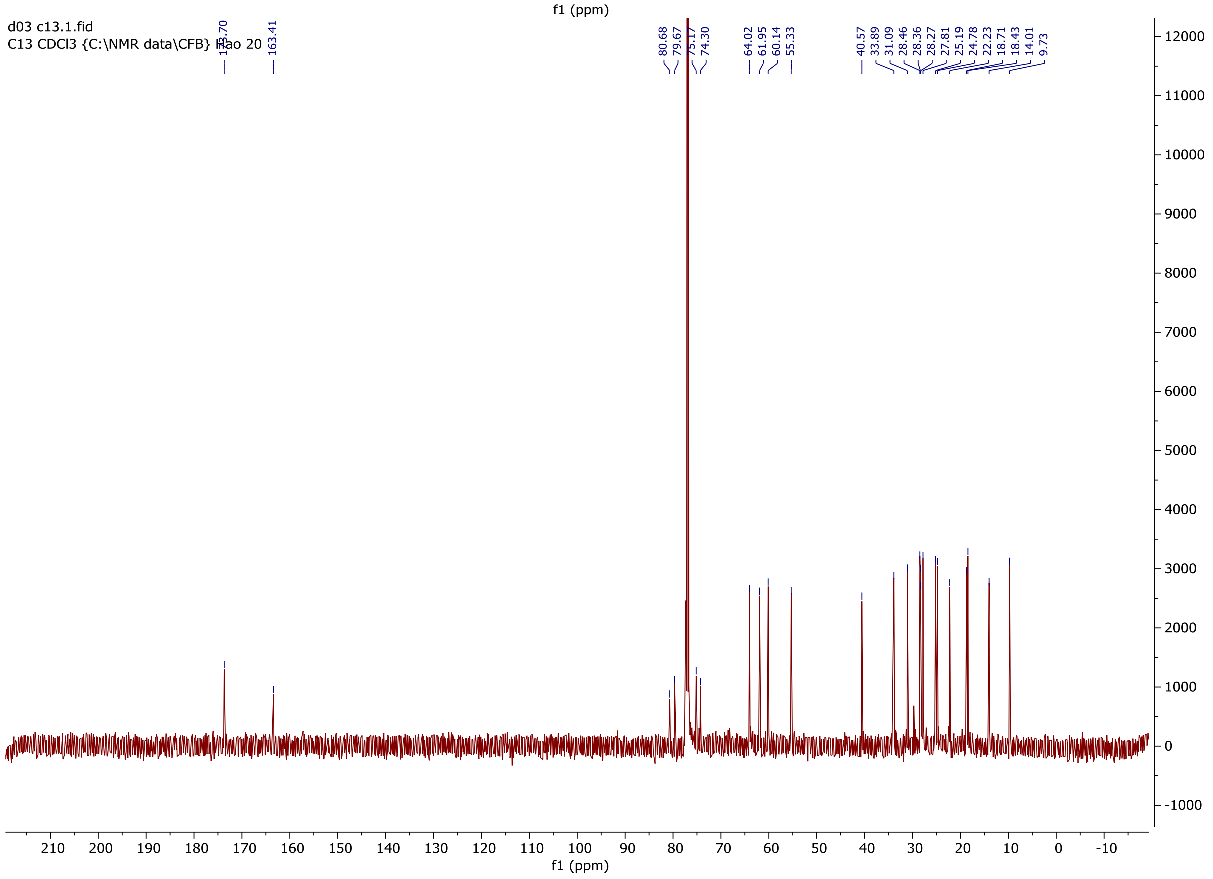
d03 c13.1.fid
C13 CDCl3 {C:\NMR data\CFB} Hao 20
163.41
80.68
79.67
75.17
74.30
64.02
61.95
60.14
55.33
40.57
33.89
31.09
28.46
28.36
28.27
27.81
25.19
24.78
22.23
18.71
18.43
14.01
9.73
f1 (ppm)

Probe **9**

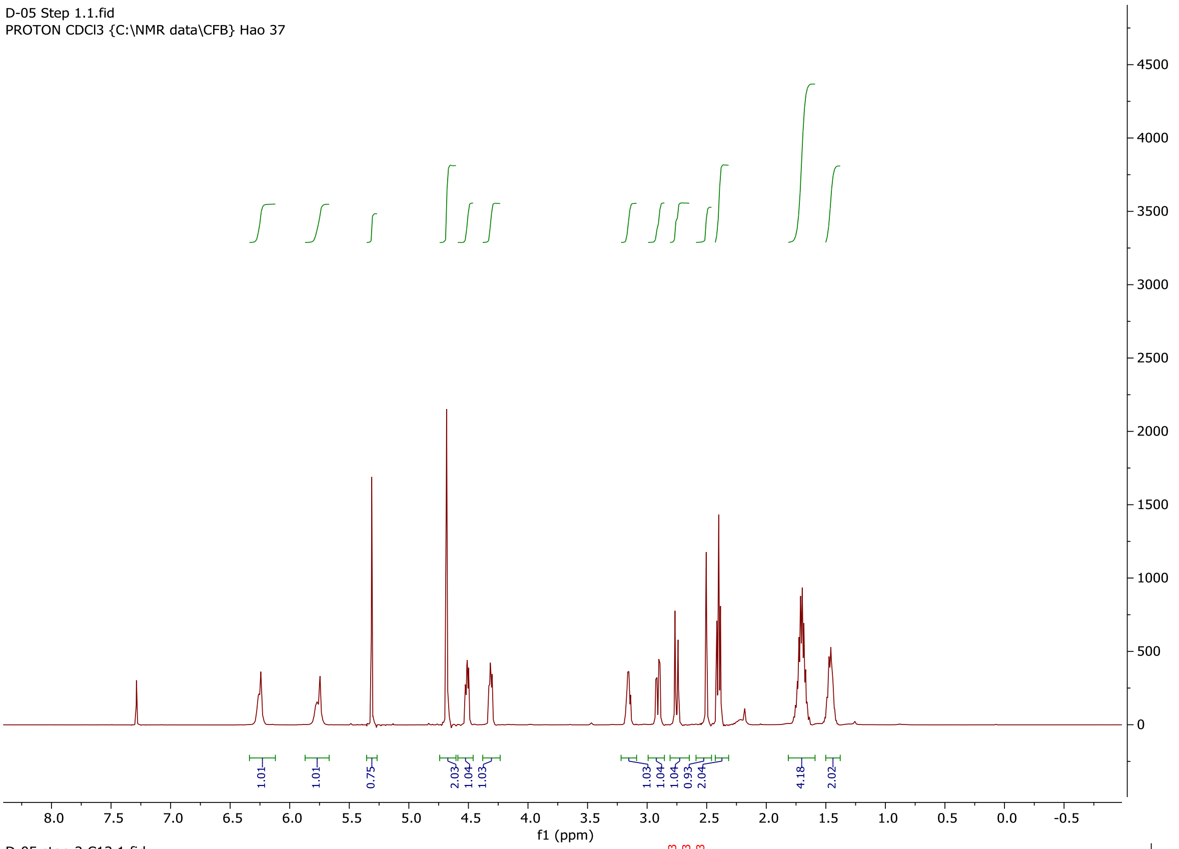
D-05 Step 1.1.fid
PROTON CDCl3 {C:\NMR data\CFB} Hao 37
1.01
1.01
0.75
2.03
1.04
1.03
1.03
1.04
1.04
0.93
2.04
4.18
2.02
f1 (ppm)

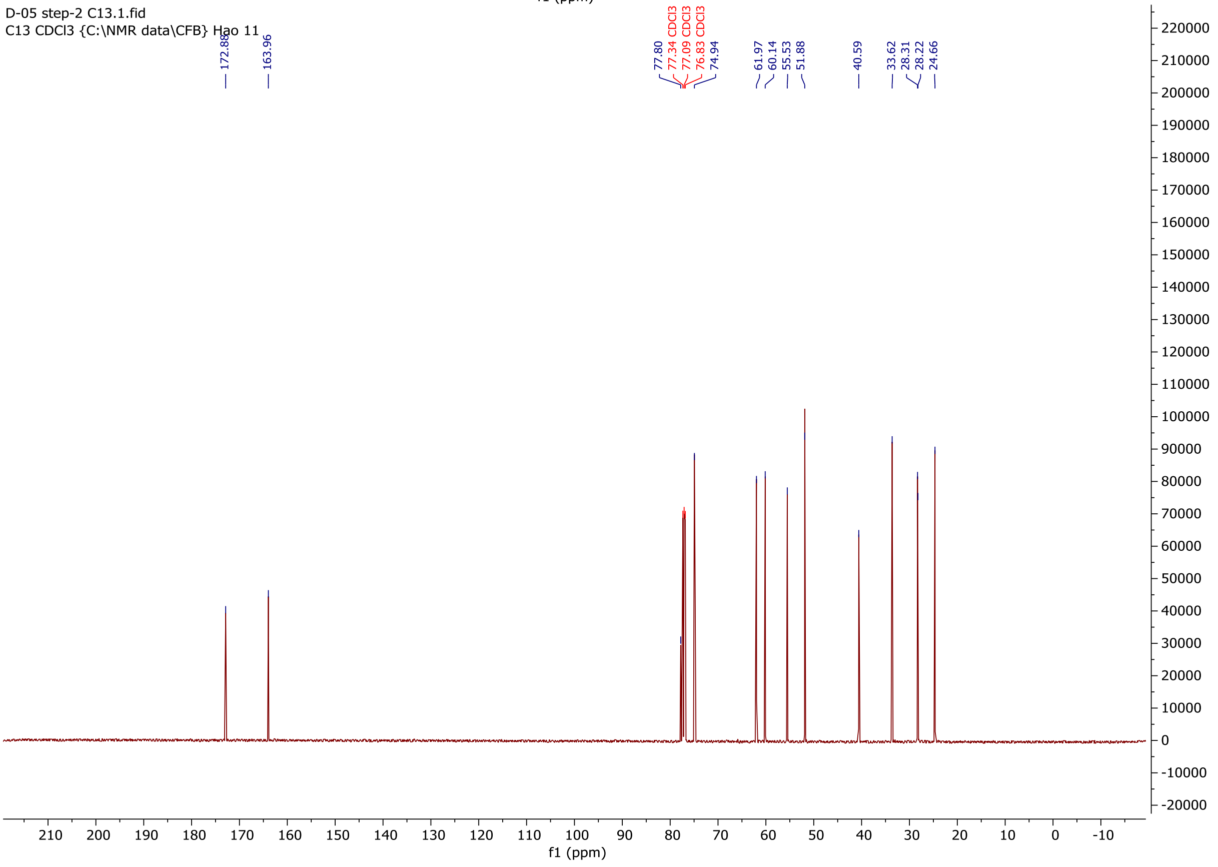
D-05 step-2 C13.1.fid
C13 CDCl3 {C:\NMR data\CFB} Hao 11
172.88
163.96
77.80
77.34 CDCl3
77.09 CDCl3
76.83 CDCl3
74.94
61.97
60.14
55.53
51.88
40.59
33.62
28.31
28.22
24.66
f1 (ppm)

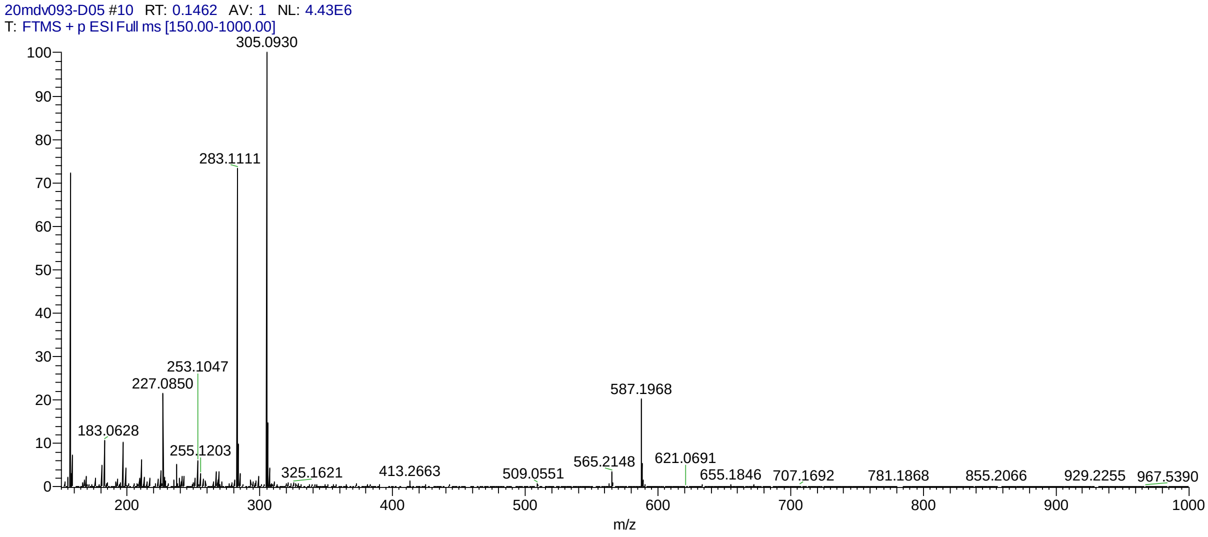
20mdv093-D05 #10 RT: 0.1462 AV: 1 NL: 4.43E6
T: FTMS + p ESI Full ms [150.00-1000.00]
305.0930
283.1111
253.1047
227.0850
183.0628
255.1203
325.1621
413.2663
509.0551
565.2148
587.1968
621.0691
655.1846
707.1692
781.1868
855.2066
929.2255
967.5390
m/z

Probe **10**

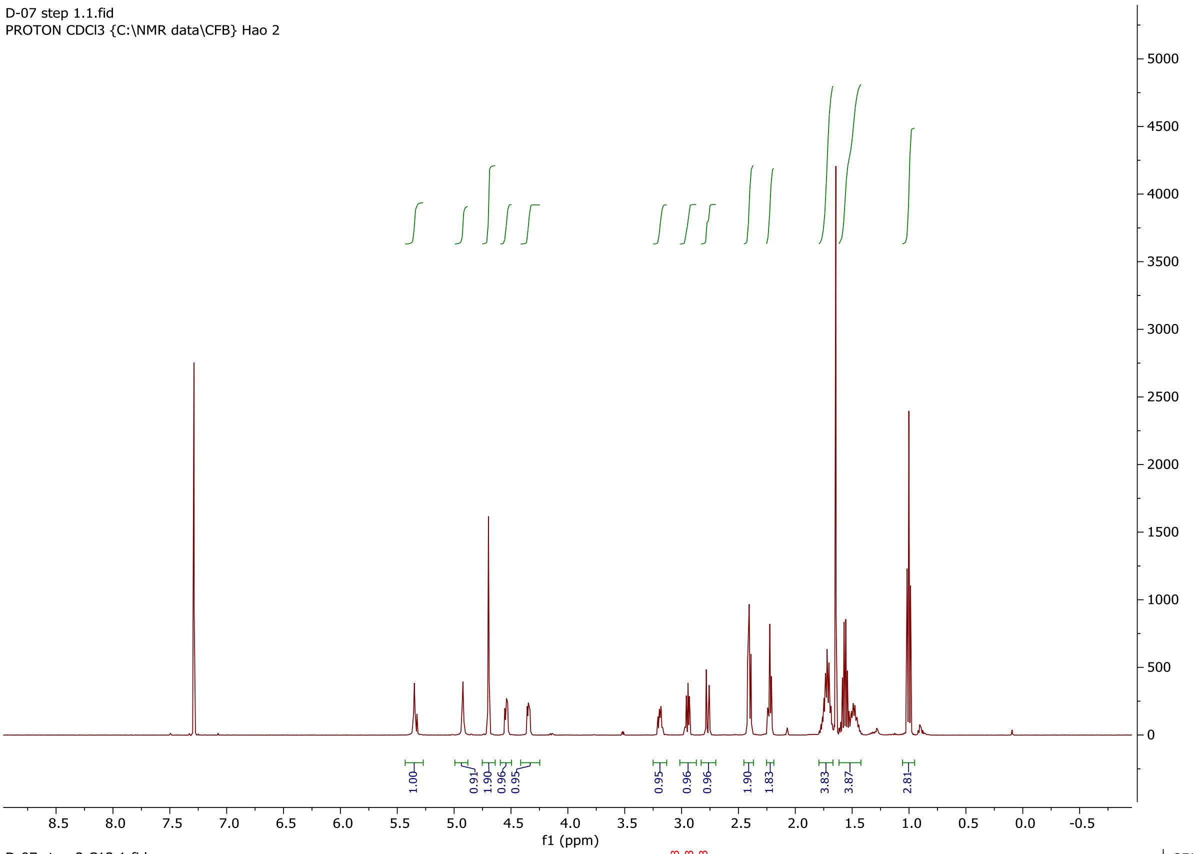
D-07 step 1.1.fid
PROTON CDCl3 {C:\NMR data\CFB} Hao 2
1.00
0.91
1.90
0.96
0.95
0.95
0.96
0.96
1.90
1.83
3.83
3.87
2.81
f1 (ppm)

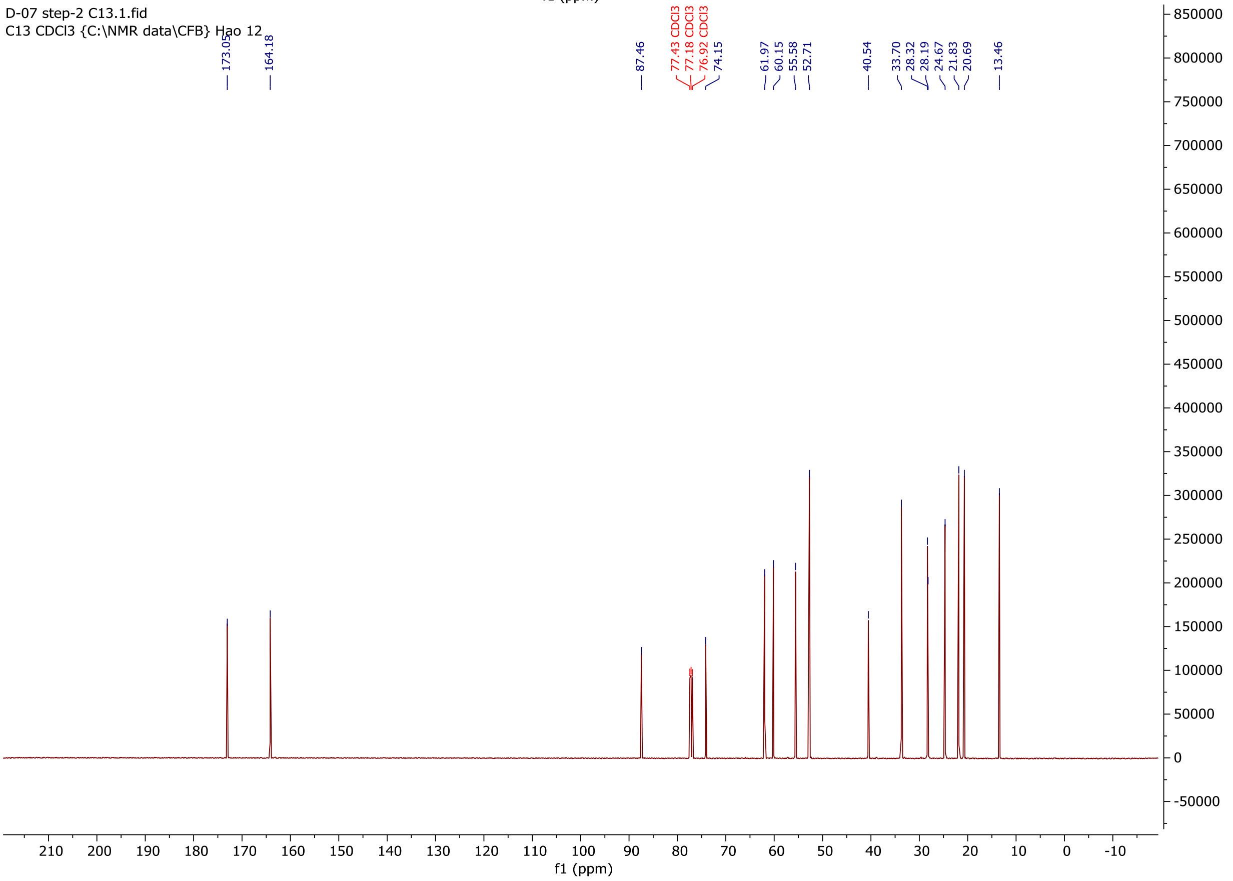
D-07 step-2 C13.1.fid
C13 CDCl3 {C:\NMR data\CFB} Hao 12
173.05
164.18
87.46
77.43 CDCl3
77.18 CDCl3
76.92 CDCl3
74.15
61.97
60.15
55.58
52.71
40.54
33.70
28.32
28.19
24.67
21.83
20.69
13.46
f1 (ppm)

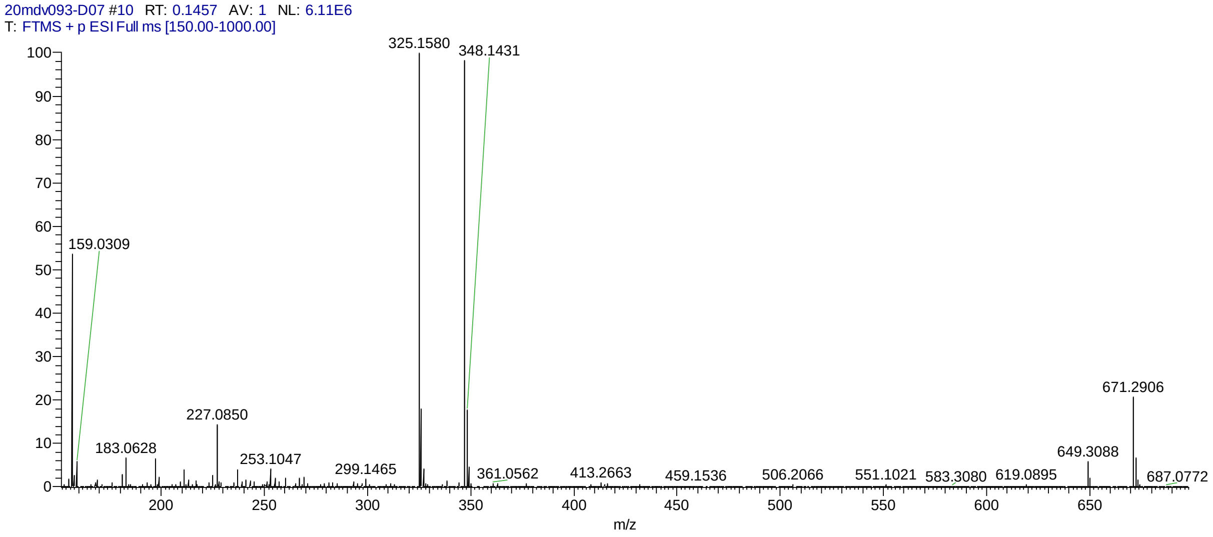
20mdv093-D07 #10 RT: 0.1457 AV: 1 NL: 6.11E6
T: FTMS + p ESI Full ms [150.00-1000.00]
325.1580
348.1431
159.0309
227.0850
183.0628
253.1047
299.1465
361.0562
413.2663
459.1536
506.2066
551.1021
583.3080
619.0895
649.3088
671.2906
687.0772
m/z

Author Contributions

Deng Chen: data curation, funding acquisition, formal analysis, investigation, writing of original draft, validation (equal)

Zhangping Xioa: data curation, funding acquisition, formal analysis, investigation, validation (equal)

Hao Guo: data curation, funding acquisition, formal analysis, investigation (equal)

Dea Gogishvili: data curation, formal analysis, investigation (supporting)

Rita Setroikromo data curation, investigation(supporting)

Petra E. van der Wouden data curation, investigation (supporting)

Frank J. Dekker: funding acquisition, project administration, writing of original draft (lead)
